# C12ORF49 inhibits ferroptosis in hepatocellular carcinoma cells via reprogramming SREBP1/SCD1-mediated lipid metabolism

**DOI:** 10.1038/s41420-025-02480-2

**Published:** 2025-04-16

**Authors:** Heng-Chao Yu, Liang Jin, Lu Bai, Yu-Jia Zhang, Zhao-Xu Yang

**Affiliations:** 1 Department of Hepatobiliary Surgery, Xijing Hospital, Air Force Medical University, Xi’an, China; 2 Department of Clinical Laboratory, Xijing Hospital, Air Force Medical University, Xi’an, China; 3 Department of Clinical Medicine, Shananxi University of Chinese Medicine, Xianyang, China

**Keywords:** Hepatocellular carcinoma, Hepatocellular carcinoma

## Abstract

Altered lipid metabolism is an emerging hallmark of cancer, which is involved in various aspects of the cancer phenotypes. C12ORF49 has recently been identified as a pivotal regulator of sterol regulatory element binding proteins (SREBPs), a family of transcriptional factors that govern lipid biosynthesis. Nevertheless, the function of C12ORF49 in human cancers has not been studied. Here, we show that C12ORF49 levels are higher in HCC tissue than in nearby non-cancerous liver tissue. Additionally, increased C12ORF49 expression is linked to poorer survival outcomes in HCC patients. Functional experiments uncovered that knockdown of C12ORF49 inhibited HCC cell survival and tumor growth by inducing ferroptosis, whereas the opposites were observed upon C12ORF49 overexpression. Mechanistically, C12ORF49 promotes SREBP1/SCD-regulated production of monounsaturated fatty acids, which inhibits ferroptosis in HCC cells. Furthermore, silencing C12ORF49 combined with Sorafenib treatment showed a synergistic effect in inducing HCC cell death. Together, our findings suggest a critical role of C12ORF49 in the evasion of ferroptosis in HCC cells, highlighting the potential of targeting C12ORF49 as a therapeutic strategy to enhance the efficacy of Sorafenib treatment in HCC.

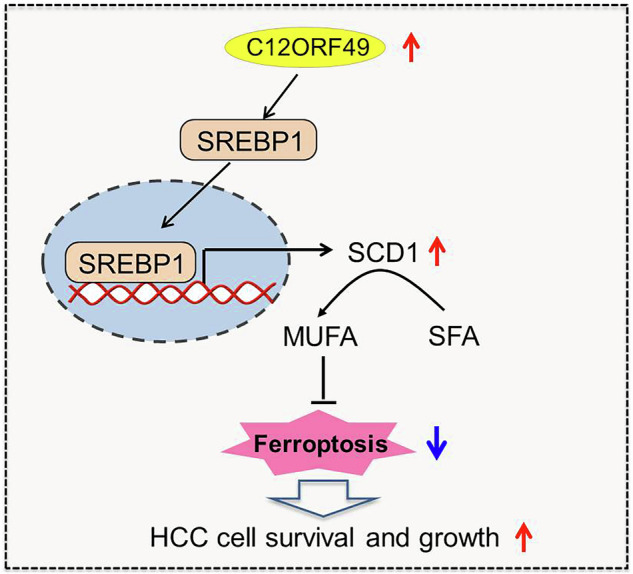

## Introduction

Despite recent advances in surgical treatment and systemic chemotherapy, hepatocellular carcinoma (HCC) patients still face a poor prognosis [1], primarily because of late-stage diagnoses [2, 3]. Sorafenib, the first FDA-approved multitargeted tyrosine kinase inhibitor, has been recommended for treating advanced HCC [4]. Recently, there has been significant focus on Sorafenib’s crucial role in promoting cell apoptosis, especially ferroptosis [5]. Nevertheless, the therapeutic effectiveness of Sorafenib is hindered by the emergence of drug resistance [6, 7]. Therefore, there is an urgent need to identify the factors that contribute to this resistance in order to enhance treatment efficacy.

Cancer cells undergo alterations in lipid metabolism, which are primarily characterized an increase in de novo lipogenesis to support their rapid growth and metastasis [8–10]. Unfortunately, how cancer cells altered lipid metabolism remain inadequately elucidated. Several critical fatty acid biosynthesis enzymes, ATP-citrate lyase (ACLY) [11], fatty acid synthase (FAS) [12], acetyl-CoA carboxylase (ACC) [13] and stearoyl-Coenzyme a desaturase 1 (SCD1) [14], as well as pivotal cholesterol biosynthesis enzymes, 3-hydroxy-3-methylglutaryl-CoA synthase 1 (HMGCS1) and 3-hydroxy-3-methylglutaryl-CoA reductase (HMGCR) [15, 16], were upregulated and facilitates the occurrence and development of multiple cancer types. These fatty acid and cholesterol synthesis enzymes are primarily regulated by SREBP1 and SREBP-2 [17, 18]. It is well known that SREBP1 and SREBP2 are produced as inactive precursors located in the cytoplasm. The proteolytic activation of SREBP1 and SREBP2 requires their translocation from the endoplasmic reticulum to the Golgi, where undergo sequential cleavage by site-1 protease (S1P) and site-2 protease (S1P), leading to their translocation to the nucleus to modulate the expressions of target genes [18]. Recently, C12ORF49 was reported as an important regulator of SREBP signaling by facilitating S1P activation [19]. Ablation of C12ORF49 resulted in diminished maturation of SREBP and reduced expression of its target genes [20, 21]. However, the role of C12ORF49 in disease progression and cellular metabolism in human cancers has not been extensively studied.

In this investigation, a comprehensive exploration into the clinical significance of C12ORF49 was conducted in HCC, focusing particularly on its functional contributions to the reprogramming of lipid metabolism and processes of carcinogenesis. Our findings revealed that C12ORF49 acts as a novel factor in facilitating the evasion of ferroptosis by activating SREBP1/SCD1-mediated lipogenesis in HCC cells. Notably, silencing of C12ORF49 rendered HCC cells more susceptible to Sorafenib treatment, underscoring the potential of C12ORF49 knockdown as a promising therapeutic strategy to improve the efficiency of Sorafenib in HCC therapy.

## Results

### C12ORF49 expression is significantly elevated and its higher level predicts poor patients’ survival in HCC

**Fig. 1:**
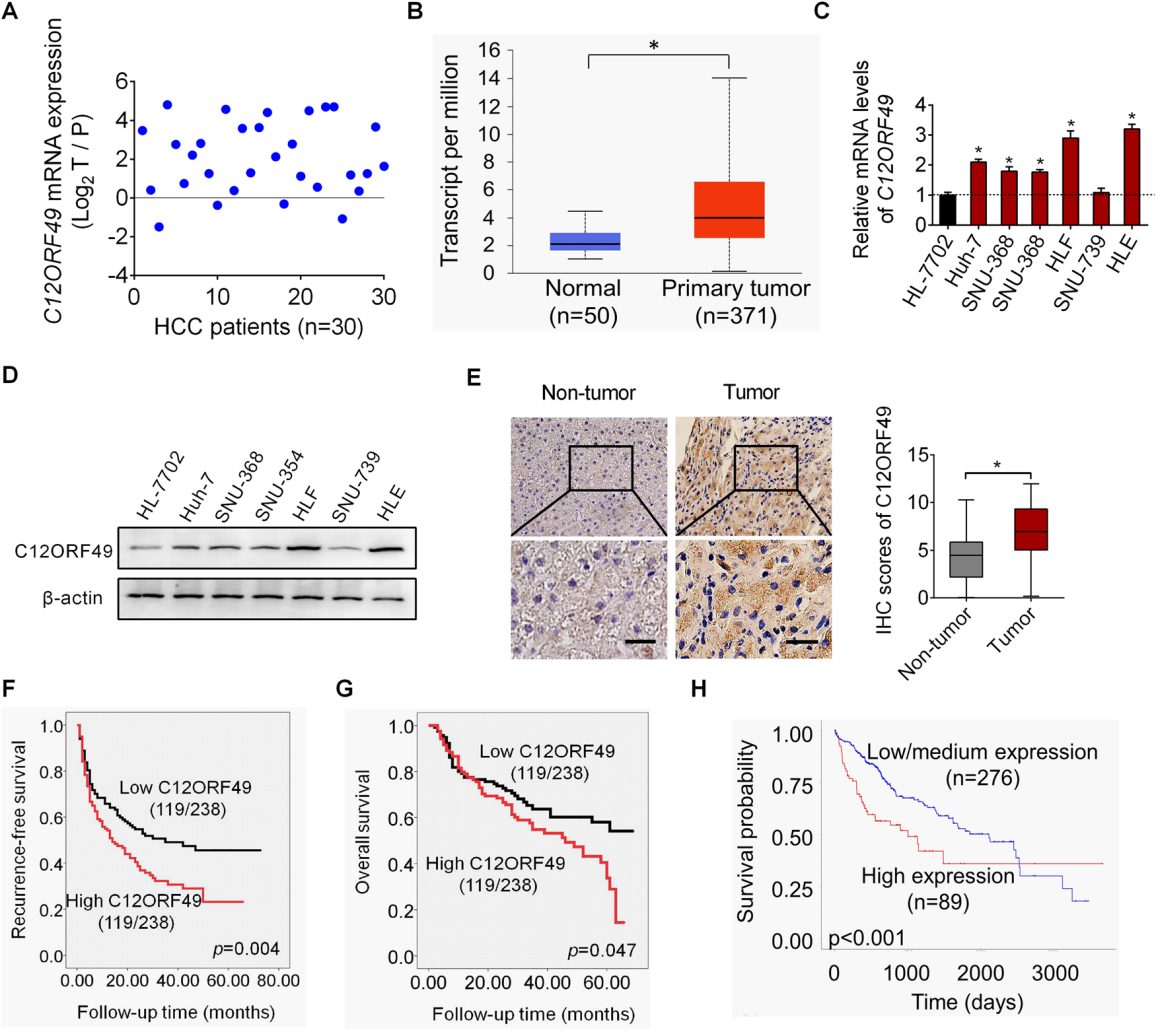
C12ORF49 expression is significantly elevated and its higher level predicts poor patients’ survival in HCC. **A** qRT-PCR assay for C12ORF49 expression in paired cancerous and adjacent non-cancerous tissues from a cohort of 30 HCC patients. **B** UALCAN analysis was conducted to compare C12ORF49 expression in human HCC tissues versus non-tumorous liver tissues. C12ORF49 expression was assessed in indicated cells through qRT-PCR (**C**) and western blot (**D**) assays. **E** Immunohistochemical (IHC) staining of C12ORF49 was performed on a larger cohort of 238-paired cancerous and adjacent non-cancerous tissues (Scale bar = 20 µm). Comparison of overall survival (**F**) in C12ORF49 high or low expression HCC patients (*n* = 238). **G** Comparison of recurrence-free survival in C12ORF49 high or low expression HCC patients (*n* = 238). **H** Prognostic relevance of C12ORF49 in HCC was evaluated through bioinformatics analysis using the UALCAN platform.

C12ORF49 expression was evaluated in HCC using qRT-PCR in paired cancerous and adjacent non-cancerous tissues of HCC (*n* = 30). C12ORF49 expressions were significantly elevated at mRNA level in cancerous tissues compared to adjacent non-cancerous tissues (Fig. 1A). Consistently, bioinformatics analysis with the UALCAN platform [22] also showed that the mRNA expression level of C12ORF49 was much higher in HCC tissue compared to normal liver tissue (Fig. 1B). We then verified that C12ORF49 expressions were higher in 5 of 6 HCC cell lines (5/6) than in the normal liver cell line HL-7702 (Fig. 1C, D). Furthermore, immunohistochemical (IHC) staining of C12ORF49 in a larger number of 238-paired cancerous and adjacent non-cancerous tissues demonstrated a significant increase in C12ORF49 expression at the protein level in cancerous tissues of HCC compared to adjacent non-cancerous tissues (Fig. 1E). An analysis of the link between C12ORF49 levels and the pathological parameters of HCC patients showed the association between increased C12ORF49 expression level and larger tumor size, while no significant correlations were observed with gender, age, metastasis, or differentiation stages (Table S3). High expressions of C12ORF49 were strongly correlated with shorter survival times for patients with HCC (Fig. 1F, G). UALCAN platform-based analysis revealed a consistent result showing that the C12ORF49 high expressing individuals experienced poorer outcomes than the C12ORF49 low-expressing ones (Fig. 1H).

C12ORF49 expressions were further assessed in other cancers with the Sangerbox 3.0 database. Significant upregulations of C12ORF49 were also found in several other types of human cancer (Supplementary Fig 1A), which are closely associated with poorer patients’ outcomes (Supplementary Fig 1B).

### C12ORF49 knockdown suppresses the viability and growth of HCC

**Fig. 2:**
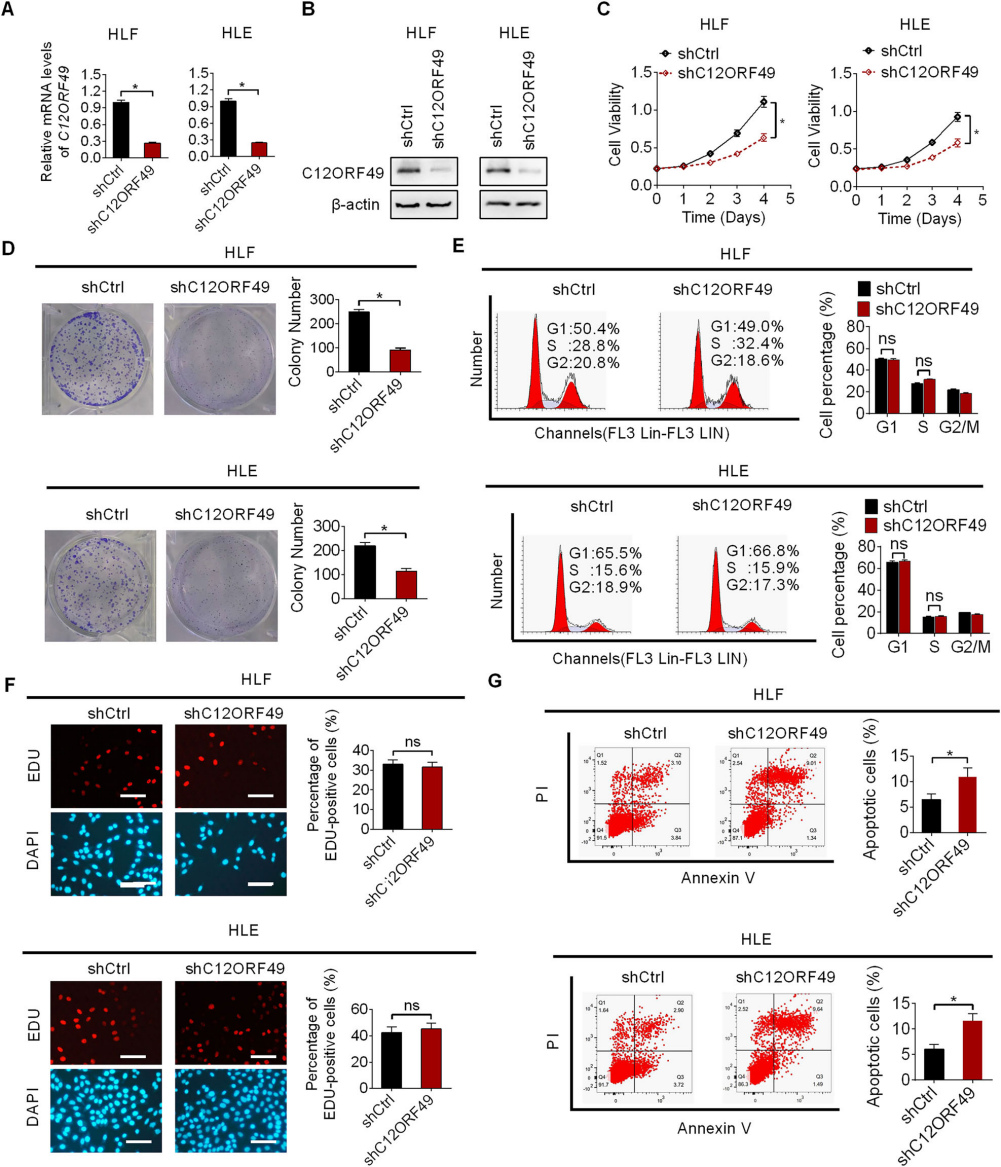
C12ORF49 knocking-down inhibited the growth of HCC cells. The effectiveness of C12ORF49 knockdown in HLF and HLE cells was validated by qRT-PCR (**A**) and WB (**B**) experiments. **C**, **D** The impact of C12ORF49 knockdown on proliferation capabilities of HLF and HLE cells were assessed by MTS and colony assays. **E**, **F** C12ORF49 knockdown on cell cycle distribution was assessed via flow cytometry and EdU incorporation assays (Scale bar = 20 µm). **G** Cell apoptosis was analyzed upon C12ORF49 knockdown. Values are expressed as mean ± SEM from three individual experiments (n = 3).

To evaluate the role of C12ORF49 in HCC, we performed knockdown of C12ORF49 in two HCC cell lines (HLF and HLE), both of which are characterized by high expression levels of C12ORF49 (Fig. 2A, B). MTS and colony formation experiments revealed that knockdown of C12ORF49 reduced both the viability and colony-forming ability of HLF and HLE cells (Fig. 2C, D). Contrary to our initial hypothesis that C12ORF49 silencing would induce cell cycle arrest, we did not observe any significant alterations in cell cycle distribution between G1/G0, S, and G2/M phase in HLF and HLE cells (Fig. 2E). In line with this observation, EdU incorporation assay indicated that knockdown of C12ORF49 has no significant effect on the percentage of cells undergoing DNA synthesis (Fig. 2F). However, C12ORF49 silencing resulted in a substantial elevation of apoptosis of HLF and HLE cells (Fig. 2G), suggesting that C12ORF49 silencing may suppress HCC cell viability and growth through increasing cell apoptosis.

We then evaluated the influence of C12ORF49 on the metastatic behavior of HLF and HLE cells. The results showed no notable alterations in metastatic capacities of HLF and HLE cells upon the t knockdown of C12ORF49 (Fig. S2A, B). Additionally, epithelial-mesenchymal-transition (EMT) assessment revealed no significant changes in the expression of epithelial and mesenchymal markers (Fig. S2C, D), further confirming that C12ORF49 does not exert a considerable effect on metastasis of HCC.

### The suppressive effect of C12ORF49 silencing on HCC growth was confirmed in mouse models

**Fig. 3:**
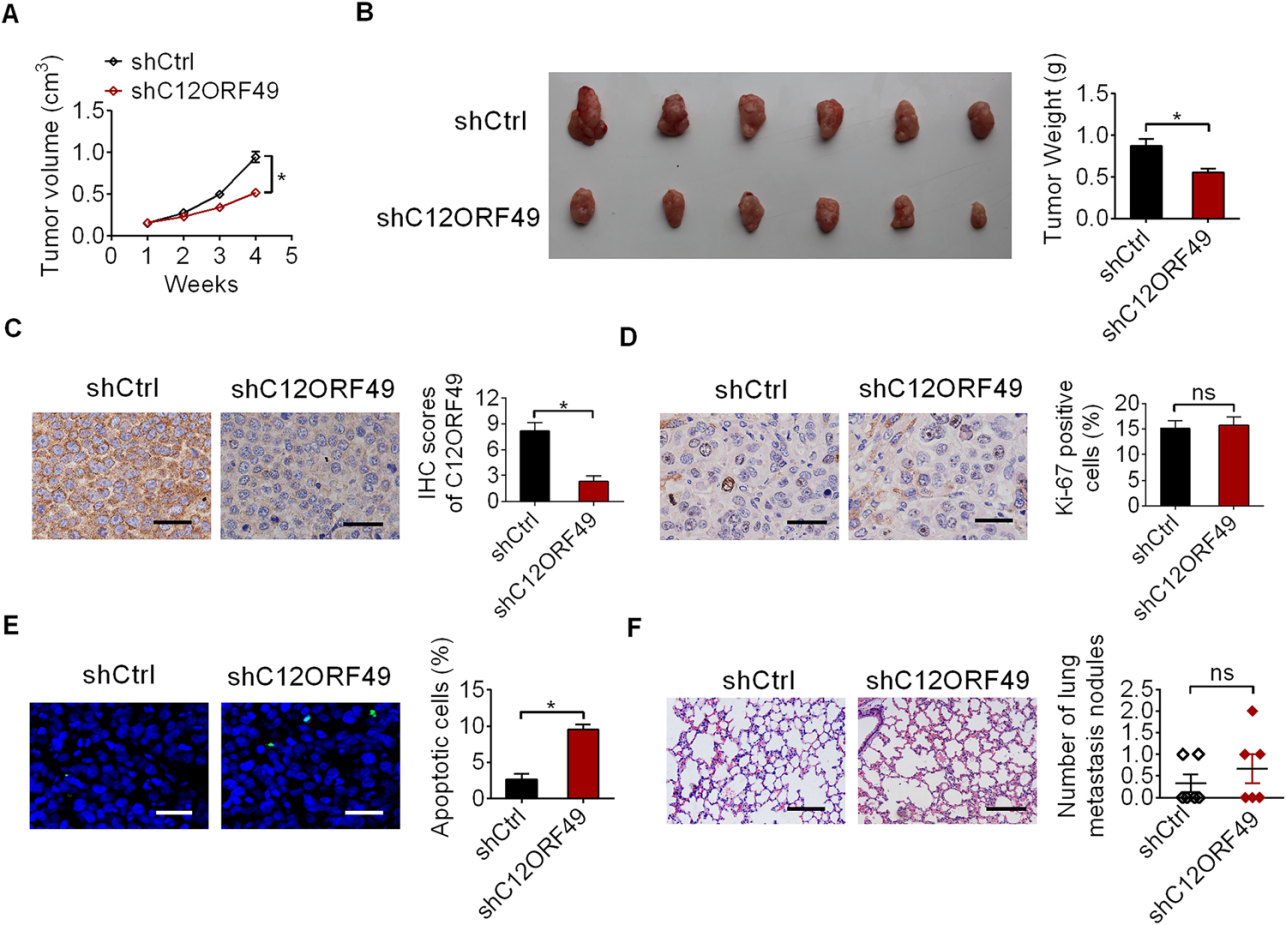
The suppressive effect of C12ORF49 silencing on HCC growth was confirmed in mouse models. **A** Subcutaneous implantation nude mice models were constructed in indicated groups. **B** Representative images and weight of tumors dissected from subcutaneous xenograft models. **C**, **D** C12ORF49 and Ki-67 expressions were assessed by IHC assay to assess the efficiency of C12ORF49 knockdown and its impact on cell proliferation (Scale bar = 50 µm). **E** TUNEL assay was employed to assess the impact of C12ORF49 knockdown on in vivo cell apoptosis (Scale bar = 50 µm). **F** The impact of C12ORF49 knockdown on in vivo lung metastatic capabilities of HLF cells was assessed in nude mice models (Scale bar = 50 µm).

Next, we validated the impact of C12ORF49 silencing on HCC growth in vivo. The results from subcutaneous implantation nude mice models demonstrated that the xenografts developed from the C12ORF49 knockdown HLF cells were much smaller than the xenografts developed from the control HLF cells (Fig. 3A, B). A notable reduction in C12ORF49 expression was observed in tumors developed from C12ORF49 silencing HLF (Fig. 3C), indicating that the observed suppressive impact on tumor growth was a result of C12ORF49 silencing. The proportion of Ki-67 positive cells remained unchanged following C12ORF49 silencing, as demonstrated by the immunohistochemistry staining assay (Fig. 3D). Conversely, cell apoptosis was increased in xenografts with C12ORF49 silencing, as determined by the TUNEL staining assay (Fig. 3E). Moreover, the metastasis in the lungs of nude mice was comparable between the C12ORF49 silencing and control groups (Fig. 3F).

### Overexpression of C12ORF49 promoted the growth of HCC cells

**Fig. 4:**
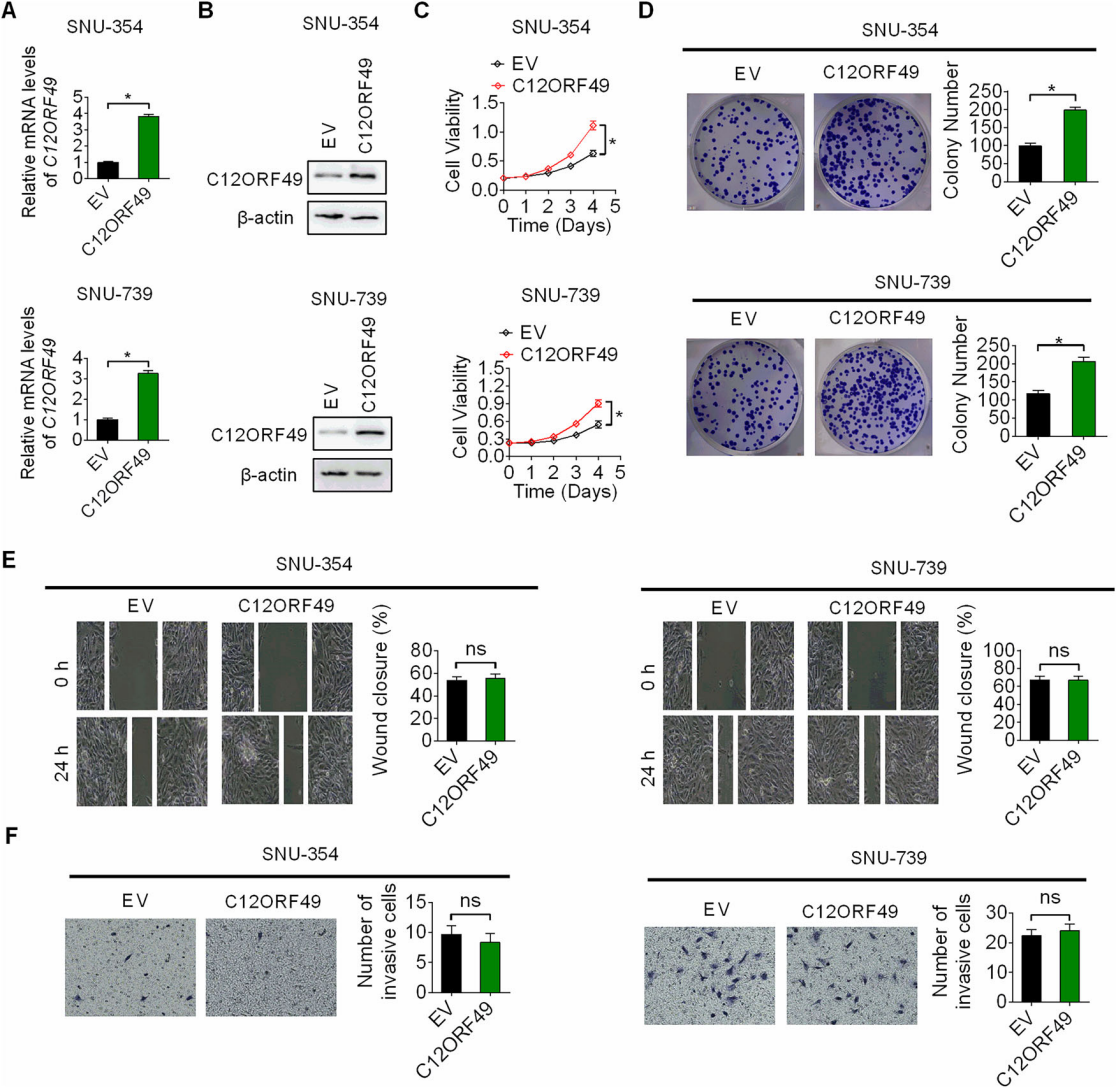
Overexpression of C12ORF49 promoted the growth of HCC. Upregulation of C12ORF49 was validated by qRT-PCR (**A**) and WB (**B**). **C**, **D** The influence of C12ORF49 overexpression on cell viability and growth was assessed by MTS and colony-forming assays. **E, F** The influence of C12ORF49 overexpression on cell migration and invasion was assessed. Values are expressed as mean ± SEM from three individual experiments (*n* = 3).

To confirm the oncogenic function of C12ORF49 in HCC, C12ORF49 was overexpressed in another two HCC cell lines (SNU-354 and SNU-739), which express low levels of C12ORF49, by transfecting them with a C12ORF49 expression plasmid. The successful elevation of C12ORF49 expression was validated in Fig. 4A, B. Our findings indicate that C12ORF49 overexpressed HCC cells had a marked increase in both viability and colony-forming capacity when compared to control (EV) cells (Fig. 4C, D). Nevertheless, no significant alterations in metastatic capabilities of these cells were noted following the upregulation of C12ORF49 (Fig. 4E, F). These data further support C12ORF49 as a pivotal promotor of HCC growth.

### C12ORF49 promotes HCC survival by suppressing ferroptosis

**Fig. 5:**
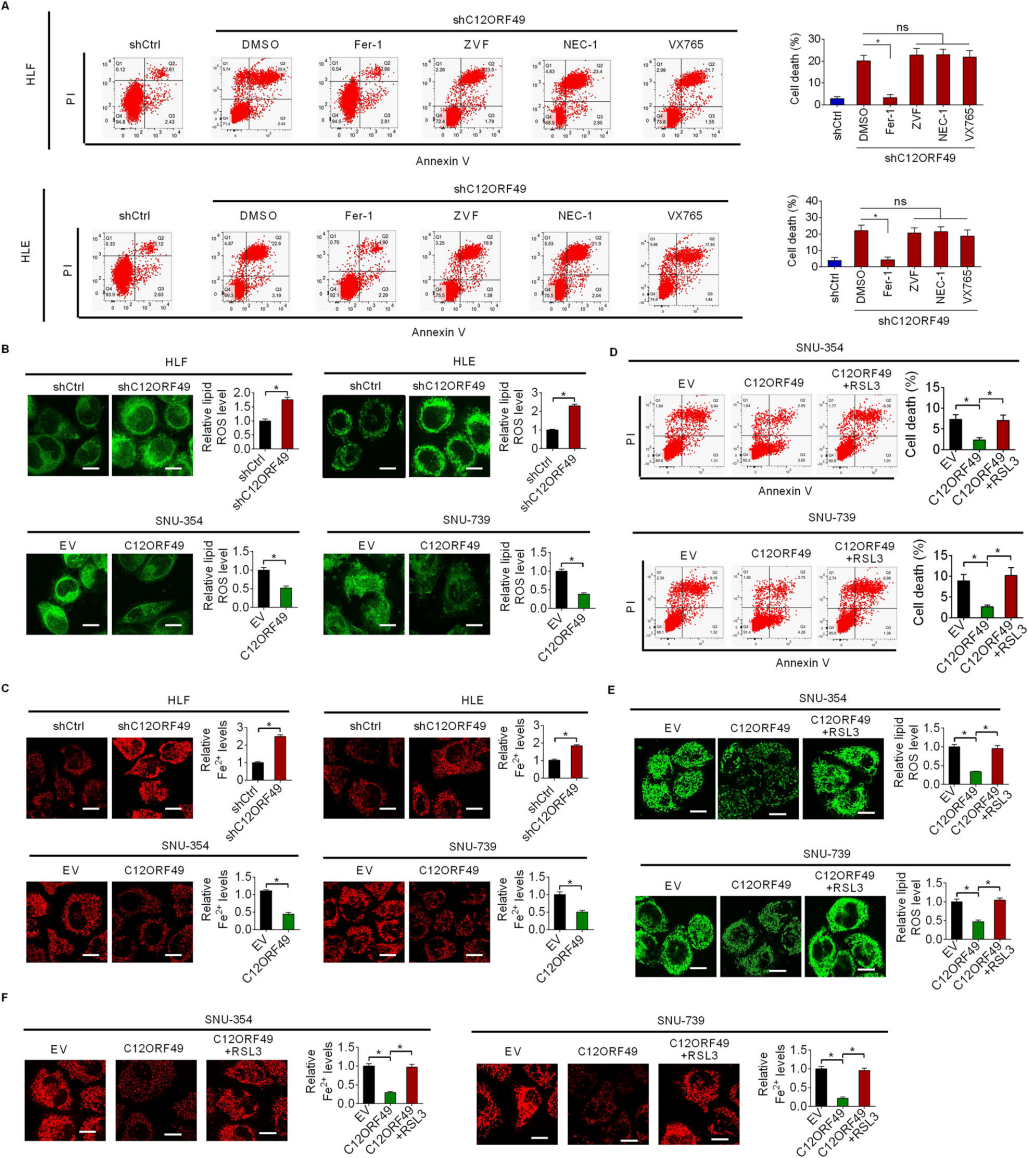
C12ORF49 promotes HCC survival by suppressing ferroptosis. **A** An assessment of cell death was conducted in HLF and HLE cells treated with inhibitors targeting distinct forms of cell death (ferroptosis inhibitor Fer-1; apoptosis inhibitor ZVF; necroptosis inhibitor NEC-1, pyroptosis inhibitor VX765). **B** Quantification of lipid reactive oxygen species (ROS) was carried out using the C11-BODIPY 581/591 staining assay (Scale bar = 20 µm). **C** Intracellular levels of Fe^2+^ were measured (Scale bar = 20 µm). Cell death (**D**) and concentrations of lipid ROS (**E**) and Fe^2+^ (**F**) were measured in SNU-354 and SNU-739 cells treated with RSL3 (Scale bar = 20 µm). Values are expressed as mean ± SEM from three individual experiments (*n* = 3).

Over the past few decades, multiple forms of regulated cell death have been characterized [23]. To identify which type of cell death is modulated by C12ORF49, HLF and HLE cells were treated with specific inhibitors (Fer-1 for ferroptosis, ZVF for apoptosis, NEC-1 for necroptosis, and VX765 for pyroptosis). Only Fer-1 effectively prevented the ferroptosis triggered by C12ORF49 silencing in HLF and HLE cell (Fig. 5A). This suggests a specific inductive effect of C12ORF49 silencing on ferroptosis in HCC cells. Consistent with our expectations, C12ORF49 silencing markedly increased the levels of lipid ROS, a defining characteristic of ferroptosis, while enforced C12ORF49 expression resulted in a reduction of lipid ROS (Fig. 5B), as evaluated by the C11-BODIPY 581/591 staining assay. Similarly, the intracellular concentrations of Fe^2+^ were found to increase upon C12ORF49 silencing and decrease with C12ORF49 overexpression (Fig. 5C). Moreover, the suppression of cell death and the reductions of lipid ROS and Fe^2+^ levels attributed to C12ORF49 overexpression were significantly reversed by treatment with RSL3 (a ferroptosis inducer) (Fig. 5D–F), further supporting the fact that C12ORF49 promotes HCC cell survival by inhibiting ferroptosis.

### C12ORF49 enhances lipogenesis by activating SREBP1/SCD1 signaling in HCC cells

**Fig. 6:**
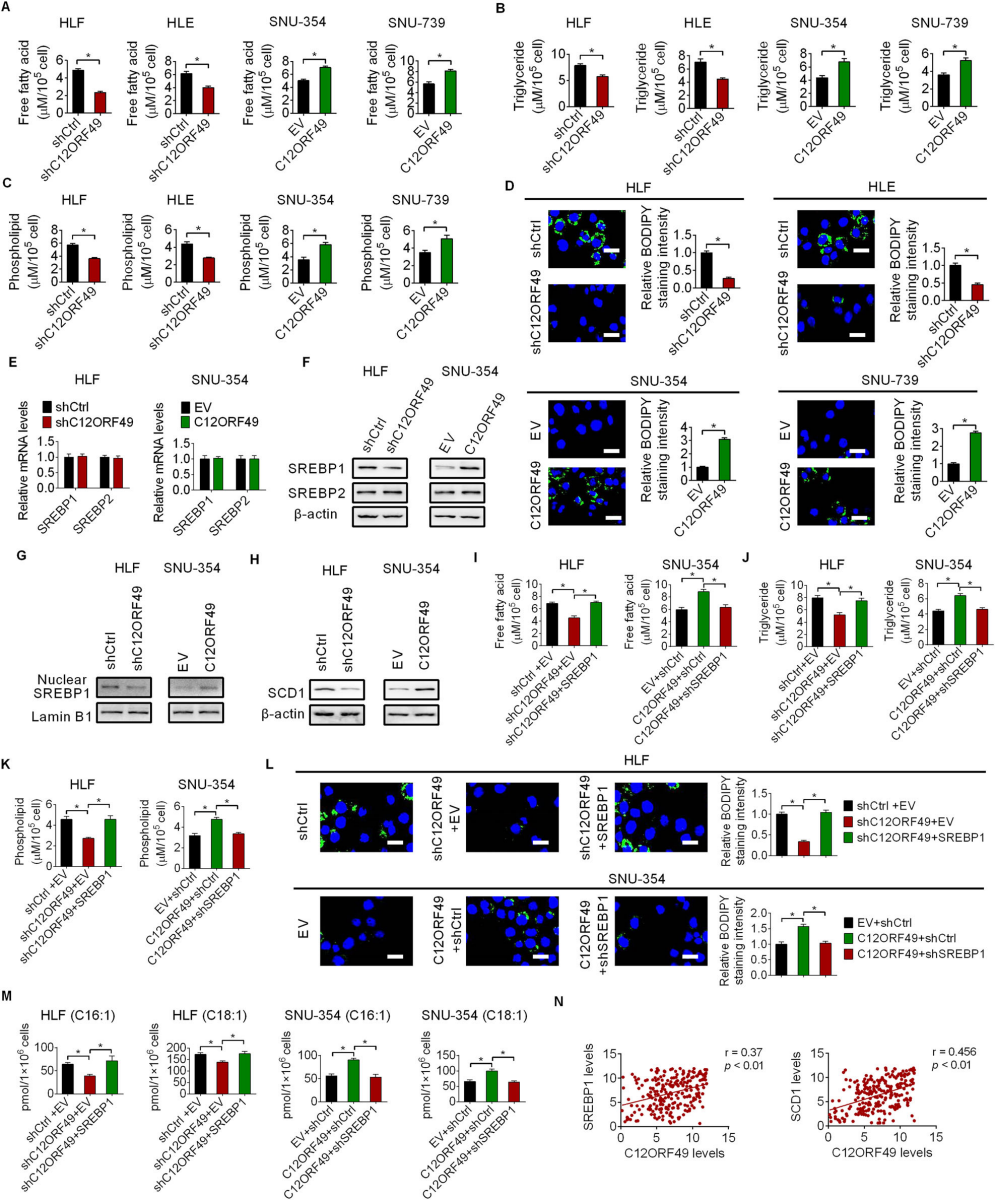
C12ORF49 enhances lipogenesis by activating SREBP1/SCD1 signaling. Concentrations of free fatty acid (FFA) (**A**), triglyceride (TG) (**B**) and phospholipids (PL) (**C**) were quantified. **D** Lipophilic BODIPY 493/503 staining assay was conducted to detect the levels of neutral lipids (Scale bar = 20 µm). The influence of C12ORF49 on the expression levels of SREBP1 and SREBP2 was evaluated by qRT-PCR (**E**) and WB (**F**) assays. **G** WB assay for the impact of C12ORF49 on the nuclear SREBP1 level. **H** WB assay was conducted for the impact of C12ORF49 on the expression of SCD. FFA (**I**), TG (**J**) and PL (**K**) levels were detected in HCC cells with indicated treatment. **L** Lipophilic BODIPY 493/503 staining assay was conducted to evaluate the neutral lipids content (Scale bar = 20 µm). **M** The contents of palmitoleic (C16:1) and oleic (C18:1) acids were detected in HCC cells with indicated treatment. **N** IHC staining assays for correlations between the expressions of C12ORF49 and SREBP1 and SCD1 in HCC tissues. Values are expressed as mean ± SEM from three individual experiments (*n* = 3).

Investigations have highlighted C12ORF49 as an important factor in regulating SREBP-mediated lipid metabolism [21]. In this context, we examined the influence of C12ORF49 on the reprogramming of lipid metabolism in HCC. As shown in Fig. 6A–C, significantly decreased the levels of intracellular free fatty acid, triglyceride and phospholipids in HLF and HLE cells following the knockdown of C12ORF49, while the contents of these lipids were all increased when C12ORF49 was overexpressed. Additionally, the BODIPY 493/503 staining assay demonstrated that knockdown of C12ORF49 resulted in a noticeable reduction in neutral lipid content, whereas its overexpression resulted in elevated neutral lipid levels in HCC cells (Fig. 6D). SREBPs consist of SREBP1 involved in fatty acid biosynthesis and SREBP2 involved in cholesterol biosynthesis, are essential regulators of lipid metabolism by transcriptionally modulating various enzymes [18]. Given the reported influence of C12ORF49 in lipid metabolism through SREBPs [20], we assessed the role of SREBPs in C12ORF49-induced lipogenesis in HCC cells. Detection of SREBP1 and SREBP2 expressions revealed that only the protein level of SREBP1 was significantly down- or upregulated when C12ORF49 was silenced or upregulated, with no corresponding changes observed in mRNA levels (Fig. 6E, F). Similar to the observed alterations in total SREBP1 protein levels, the nuclear localization of SREBP1, which represents its active form, was also significantly down- or up-regulated when C12ORF49 was silenced or upregulated (Fig. 6G), indicating the activation of SREBP1 by C12ORF49 in HCC cells. After that, the effect of C12ORF49 on the expression levels of critical lipogenesis enzymes (ACLY, ACC, FASN and SCD1) was determined. The findings indicated that SCD1, a major enzyme required for monounsaturated fatty acid (MUFA) biosynthesis, was positively regulated by C12ORF49 in HCC cells (Figs. 6H and S3A). We then assessed whether C12ORF49 promotes fatty acid synthesis via activating SREBP1/SCD1 in HCC cells. Notably, the suppression of fatty acid synthesis by C12ORF49 knockdown was significantly reversed by SREBP1 overexpression, whereas SREBP1 knockdown attenuated the effects of enforced C12ORF49 expression on fatty acid synthesis (Fig. 6I–L). SCD1 catalyzes the rate-limiting step in the synthesis of MUFA, including palmitoleic and oleic acid. Our results further demonstrated that that the levels of palmitoleic (16:1) and oleic acid (18:1) were significantly down- or up-regulated when C12ORF49 was silenced or upregulated in HCC cells. Restoring SCD1 expression clearly reversed these effects on palmitoleic (C16:1) and oleic (C18:1) acids (Fig. 6M). Furthermore, IHC staining assays revealed significant positive correlations between the expressions of C12ORF49 and SREBP1 and SCD1 in HCC tissues (Fig. 6N and Fig. S3B). Together, these results suggest that upregulation of C12ORF49 enhances lipogenesis in HCC cells by activating SREBP1/SCD1 signaling.

### C12ORF49 suppresses ferroptosis by activating SREBP1/SCD1-mediated biogenesis of monounsaturated fatty acids

**Fig. 7:**
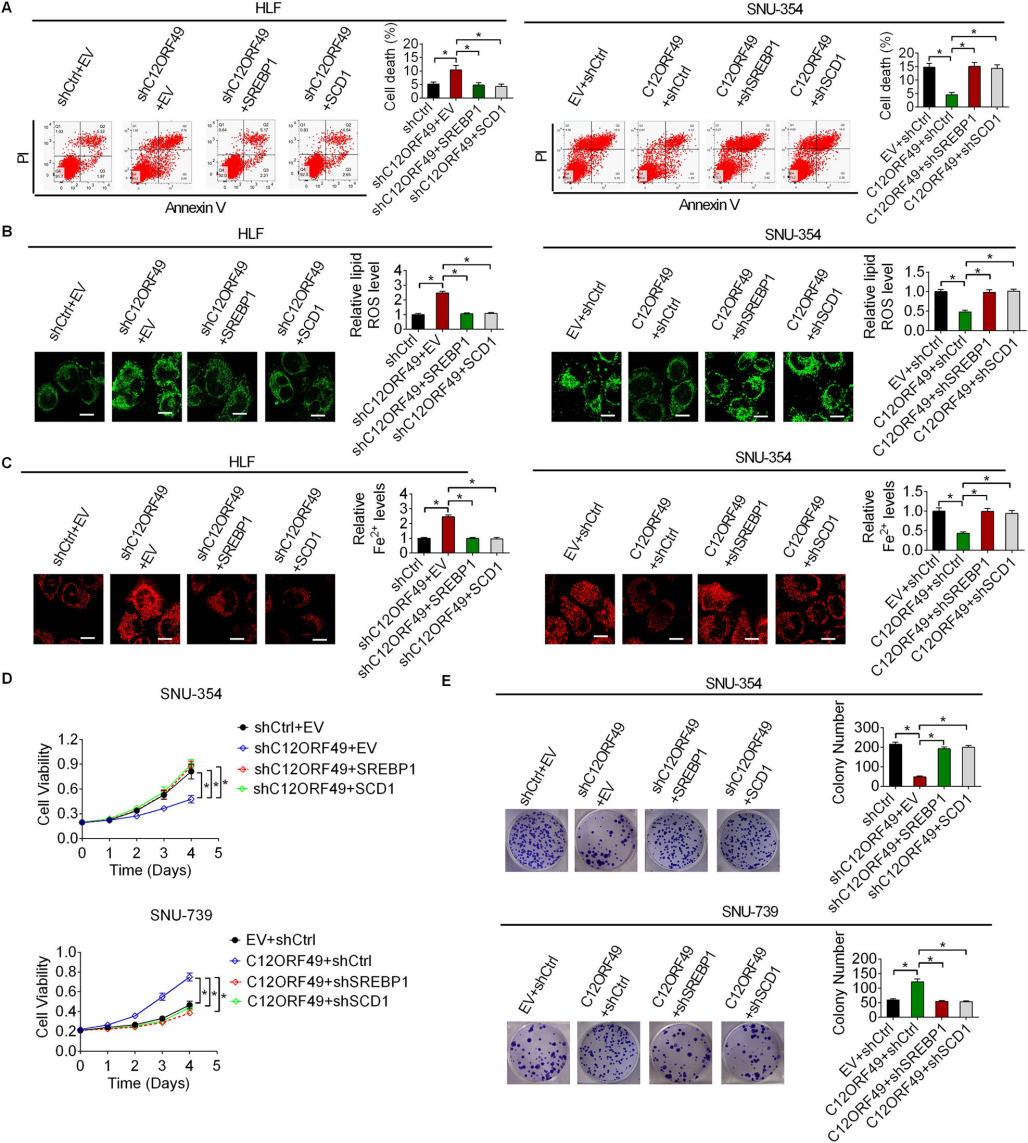
C12ORF49 suppresses ferroptosis by activating SREBP1/SCD1-mediated biogenesis of monounsaturated fatty acids. **A** Cell death was evaluated in HCC cells under indicated treatment. Quantifications of lipid ROS (**B**) and Fe^2+^ (**C**) (Scale bar = 20 µm). **D**, **E** Cell viability and growth were assessed using MTS and clonogenic assays. Values are expressed as mean ± SEM from three individual experiments (*n* = 3).

Given that a recent investigation has suggested the link between the activation of SREBP1/SCD1 and ferroptosis inhibition in synovial fibroblasts cells [24], we hypothesized that C12ORF49 could inhibit ferroptosis and promote HCC growth by activating SREBP1/SCD1-mediated lipogenesis. In Fig. 7A–C, either upregulation of SREBP1 or SCD1 markedly attenuated C12ORF49 silencing-induced ferroptosis, while silencing either SREBP1c or SCD1 significantly counteracted the inhibitory effects of C12ORF49 overexpression on ferroptosis. In agreement, the enforced expression of either SREBP1 or SCD1 significantly mitigated the suppressive effects of C12ORF49 silencing on HCC cell viability and colony formation, whereas reduction of either SREBP1c or SCD1 clearly diminished the promotive effects of C12ORF49 upregulation on HCC growth in SNU-354 and SNU-739 cells (Fig. 7D, E). These findings indicate that C12ORF49 suppresses ferroptosis and thus promotes HCC growth through the activation of SREBP1/SCD1-regulated lipogenesis.

### C12ORF49 silencing enhances the efficiency of sorafenib treatment on the suppression of HCC growth and induction of ferroptosis

**Fig. 8:**
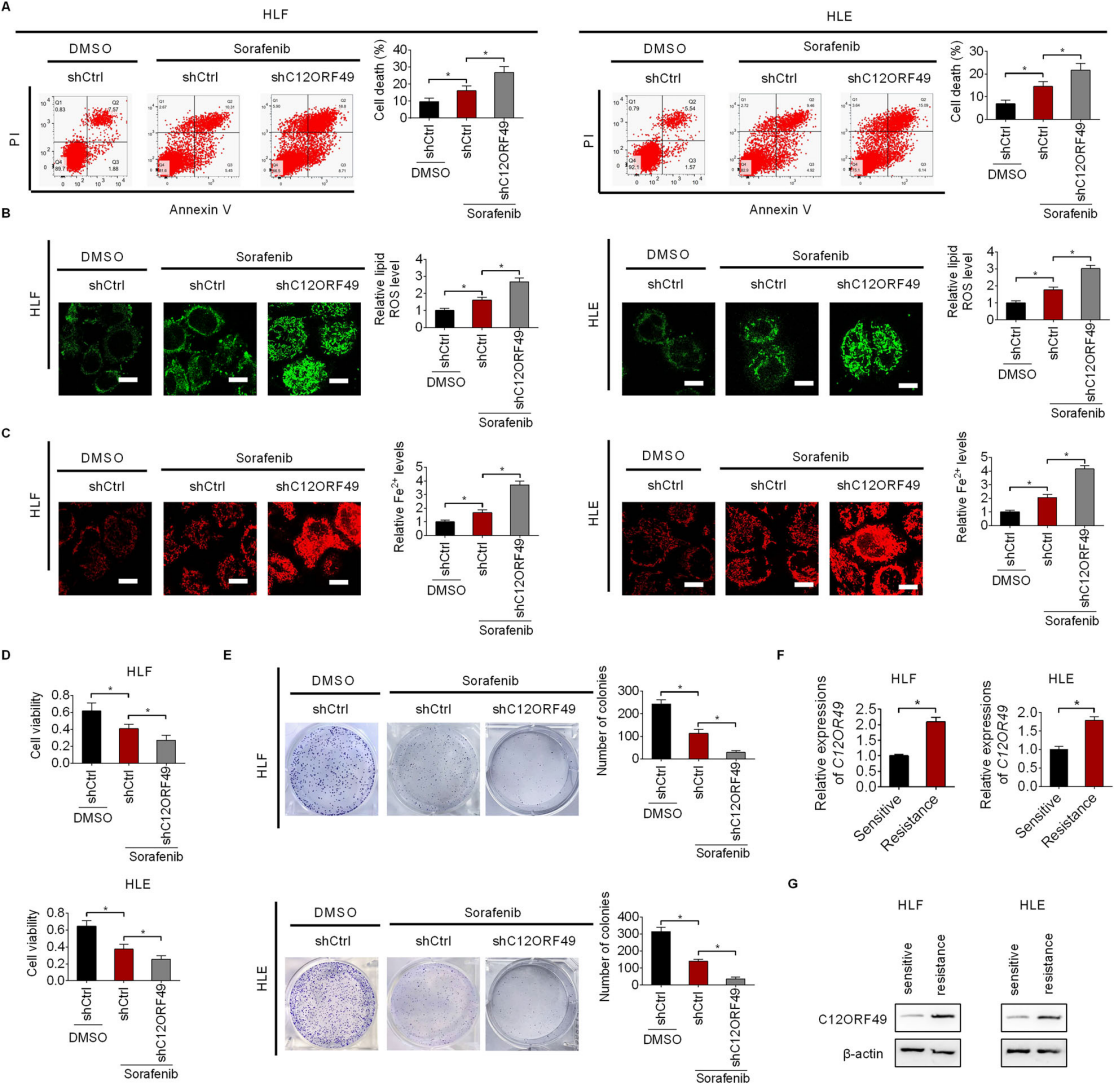
C12ORF49 silencing enhances the efficiency of sorafenib treatment on the suppression of HCC growth and induction of ferroptosis. **A** Cell death in HLF and HLE cells was evaluated. Sorafenib (10 μM). Quantifications of lipid ROS (**B**) and Fe2^+^ (**C**) in HLF and HLE cells (Scale bar = 20 µm). MTS (**D**) and colony (**E**) assays in HLF and HLE cells. **F**, **G** qRT-PCR and WB assays were conducted to determine C12ORF49 expression in Sorafenib-resistant or -sensitive HLF and HLE cells. Values are expressed as mean ± SEM from three individual experiments (*n* = 3).

Sorafenib, a multikinase inhibitor, has been used for targeted therapy in advanced patients with HCC [4]. Nonetheless, the survival benefits associated with Sorafenib are often limited owing to the drug resistance [6]. Considering the role of sorafenib in ferroptosis induction [25], we investigated whether the silencing of C12ORF49 could enhance the anti-tumor activity of sorafenib. Flow cytometry analysis showed that C12ORF49 silencing obviously increased sorafenib treatment-induced cell death (Fig. 8A). Corresponding alterations were observed in the levels of lipid ROS and Fe^2+^ (Fig. 8B, C), suggesting that C12ORF49 silencing enhances the anti-tumor efficacy of Sorafenib. Additionally, the inhibitory effects of Sorafenib on HCC cell growth were significantly augmented by C12ORF49 silencing (Fig. 8D, E). Furthermore, the expression of C12ORF49 was higher in Sorafenib-resistant HCC cells than the parental Sorafenib-sensitive counterparts (Fig. 8F, G). Together, these findings indicate that C12ORF49 may promote Sorafenib resistance in HCC and its silencing could serve as a strategy to enhance the efficacy of Sorafenib treatment.

## Discussion

Elevated de novo lipogenesis has been documented in multiple cancer types, which contributes to their malignant progression through multiple mechanisms [8, 10]. SREBPs constitute a family of transcription factors that modulate the expression of enzymes involved in de novo lipogenesis [18]. Recently, Acyl-CoA thioesterases 9 (C12ORF49) has been identified as a pivotal regulator in the maturation of SREBP by facilitating the activation of S1P [19]. Nonetheless, the involvement of C12ORF49 in cancer has yet to be thoroughly investigated. Here in the current study, C12ORF49 expression was aberrantly elevated in hepatocellular carcinoma (HCC) and was associated with poorer prognosis in patients with HCC. In concordance with this, bioinformatics analysis using the UALCAN platform [22] showed that C12ORF49 was also elevated in HCC, which predicts poor survival in HCC patients. Furthermore, a pan-cancer analysis conducted using the online Sangerbox 3.0 revealed that the upregulation of C12ORF49 is a frequent event across most human cancer types, implying a pivotal role for C12ORF49 in human cancers, including HCC.

Our loss- and gain-of-function explorations revealed that C12ORF49 enhances the viability and clonogenic potential of HCC cells, while exerting minimal impact on cell metastatic ability. In keeping with our in vitro findings, xenograft tumor models indicated that C12ORF49 knockdown inhibited the tumorigenicity of HCC. Contrary to our initial hypothesis that C12ORF49 would accelerate cell cycle progression, we did not detect any significant alterations in cell cycle distribution. Instead, C12ORF49 knockdown resulted in increased apoptosis in HCC, suggesting that C12ORF49 may facilitate HCC growth by suppressing apoptosis. Besides, a higher proportion of apoptotic cells were also identified in xenograft tumor tissues derived from C12ORF49 silencing, further supporting the notion that C12ORF49 promotes HCC growth by inhibiting apoptosis. Moreover, we demonstrated that C12ORF49 suppressed HCC cell death by decreasing ferroptosis, a special form of cell death mainly caused by iron-associated lipid peroxidation, which has been proposed as a promising strategy for cancer treatment.

Considering the established role of Acyl-CoA thioesterases 9 (C12ORF49) as a pivotal regulatory element of SREBPs [20], which are essential transcriptional factors regulating the enzymes involved in de novo lipogenesis [18], which plays important roles in tumorigenesis and cancer development [18], the influences of C12ORF49 knockdown or overexpression on SREBPs and lipid metabolism were explored. Our findings revealed that the intracellular levels of lipid contents, including FFA, TG and PL, were all significantly down- or up-regulated when C12ORF49 was silenced or upregulated. Additionally, we observed that SREBP1 and its downstream stearoyl-CoA desaturase 1 (SCD1) signaling were activated by C12ORF49 in HCC cells. SCD1 serves as a major enzyme in conversing saturated fatty acid (SFA) to monounsaturated fatty acid (MUFA), and multiple studies have linked the upregulation of SCD1 to the aggressiveness of cancer [26]. Here, we found that C12ORF49 suppressed ferroptosis by activating the SREBP1/SCD1-mediated biosynthesis of monounsaturated fatty acids. This is in line with the findings from a recent study, which demonstrated that activation of SREBP1/SCD1 signaling could inhibit ferroptosis in synovial fibroblasts cells [24]. Similarly, it was reported in colorectal cancer that aspirin promotes RSL3-induced ferroptosis by inhibiting SREBP-1/SCD1-regulated lipogenesis [27]. These observations imply that the regulation of ferroptosis by SREBP1/SCD1 signaling occurs in both physiological and pathological contexts.

Sorafenib, a multikinase inhibitor, has been used for targeted therapy in advanced HCC [28, 29]. However, the therapeutic efficacy and survival benefit of Sorafenib remains limited due to the emergence of drug resistance [5]. Our results indicated an upregulation of C12ORF49 in Sorafenib-resistant HCC cells. Notably, the silencing of C12ORF49 enhanced the suppressive effects of Sorafenib on cell growth and induction of ferroptosis in HCC cells, suggesting that C12ORF49 may cause Sorafenib resistance in HCC and its silencing could represent a promising therapeutic strategy to improve the effect of Sorafenib treatment. These findings are encouraging and further in vivo animal studies will be conducted in our future study.

There are several limitations in the present study. First, the mechanism underlying the upregulation of C12ORF49 in hepatocellular carcinoma (HCC) cells remains to be explored. Second, the mechanisms linking C12ORF49 to iron homeostasis regulation in HCC cells are still unclear. Third, it remains unknown why the activation of the C12ORF49/SREBP1 pathway only transcriptionally upregulates SCD1, but not other lipogenic enzymes. Therefore, more comprehensive studies should be conducted in the future to address these issues.

Altogether, our data reveal that C12ORF49 is aberrantly upregulated in HCC and serve as a critical regulator in promoting HCC survival and growth by promoting the evasion of ferroptosis through the activation of SREBP1/SCD1-mediated MUFA biosynthesis. Additionally, our findings highlight C12ORF49 silencing as a potential therapeutic strategy to improve the effectiveness of Sorafenib in HCC.

## Materials and methods

### Clinical specimens and reagents

HCC tumor and non-tumor tissues (*n* = 268) were acquired from patients diagnosed with hepatocellular carcinoma (HCC) at the Air Force Medical University in Xi’an, China. The tissue samples were obtained with the written informed consents from all participants. The study was approved by the ethics committee of the Xijing Hospital of the Air Force Medical University (No. 20233390-1), and all methods were conducted in strict compliance with the relevant guidelines and regulations.

Fer-1 (Ferrostatin-1), an inhibitor of ferroptosis, was purchased from Sigma (Catalog No. SML0583). ZVF (Z-VAD-FMK), an inhibitor of apoptosis, was purchased from Sigma (Catalog No. V116). NEC-1 (Necrostatin-1), an inhibitor of necroptosis, was purchased from Sigma (Catalog No. N9037). VX765, an inhibitor of pyroptosis, was purchased from Selleckchem (Catalog No. S2228).

### Cell lines and transfection

The human HCC cell lines (Huh-7, SNU-368, SNU-354, HLF, SNU-739 and HLE) and normal liver cell line HL-7702 used in our present investigation were maintained in Dulbecco’s modified Eagle’s medium (DMEM) containing 10% fetal bovine serum (FBS) with an environment containing 5% CO_2_ at 37 °C. HL-7702, HLE and HLF were purchased from the Cell Bank of the Chinese Academy of Sciences (CAS). Huh-7, SNU-368, SNU-354 and SNU-739 were purchased from the American Type Culture Collection (ATCC).

Small interfering RNA (siRNA) oligonucleotides were employed to downregulate C12ORF49. The target sequences of C12ORF49 are 5′-AGGAAGGATTTGCTGGTAAATGG-3′. To generate C12ORF49 expression plasmid, the full-length of human C12ORF49 cDNA was amplified by PCR and inserted to a pcDNA3.1(+) vector. Transfection of cells with either siRNA or plasmids was conducted when they reached about 60% confluence at the help of Lipofectamine 3000 (Invitrogen) following the manufacturer’s protocol. Briefly, cells were incubated with a siRNA-lipofectamine or DNA-lipofectamine mixture in serum-free medium for 6 h at 37 °C, and then replaced with complete medium for 24 h. Successful transfection was confirmed by RT-PCR and Western blot analysis.

### Quantitative real-time PCR

RNA extraction was conducted using TRIzol reagent (Invitrogen) from HCC cell lines and frozen tissues. Following the synthesis of complementary DNA (cDNA), qPCR reaction (10 s at 95 °C, followed by 35 cycles of 95 °C for 5 s and 60 °C for 30 s) was conducted on an ABI 7900HT Real-Time PCR system (Applied Biosystems) using SYBR™ Green Universal (Thermo Fisher, Catalog No: 4309155). Relative expression of genes was calculated by 2^−ΔCt^ method with β-actin serving as an internal control. Primer sequences are available in Table S1.

### IHC staining

Tissue slides were deparaffinized using xylene and graded alcohol, and subjected to antigen retrieval in heated citrate buffer. Then, the slides were successively incubated with 3% H_2_O_2_, 3% BSA, specific primary and secondary antibodies. The signal was developed with DAB dye followed by counterstaing with hematoxylin.

Relative expression was assessed by evaluating both the intensity and the percentage of positive staining observed under a light microscope. Staining intensity was categorized and scored as 0 for negative, and 1-3 for weak-strong staining. Staining percentage was scored as 0, 1, 2, 3 and 4 for positive staining percentage of 0%, 1–25%, 26–50%, 51–75% and 76–100%. In the end, the final IHC staining score was calculated by multiplying the two aforementioned values. C12ORF49 high or low expression patients were divided by the median IHC staining score (6.98) of C12ORF49 in those patients (*n* = 238).

### Western blotting

RIPA lysis buffer (MedChemExpress, Cat. No. HY-K1001) containing protease cocktail (Roche, Catalog No. 04693116001) was used for total protein extraction from HCC cell lines. The proteins were separated by SDS/PAGE followed by transferring onto PVDF membrane (Millipore). Then, 5% non-fat milk, appropriate primary and corresponding HRP-labeled secondary antibodies were sequentially added to the membranes. A chemiluminescence detection system was utilized for results detection. The primary antibodies can be found in Table S2.

### Flow cytometry analysis for cell cycle and apoptosis

The quantifications of cell cycle and apoptosis were carried out utilizing a cell cycle distribution kit alongside an Annexin V and propidium iodide (PI) apoptosis kit, both from US Everbright Inc. Following gentle vortex, the analysis was conducted using a NovoCyte Quanteon flow cytometer (Agilent) within 1-h period. The signal is excited by a 488 nm laser, and the fluorescence signal is collected in the FL1 channel (for Annexin V) or the FL2 channel (for PI).

### Lipid content measurement

Lipid extraction was performed with the chloroform/methanol solution. Then, the levels of intracellular phospholipids (PL), triglyceride (TG), free fatty acid (FFA), and cholesterol (CL) were quantified using corresponding commercial kits, including EnzyChrom™ Phospholipid Assay Kit (Catalog No: EPLP-100), EnzyChrom™ Triglyceride Assay Kit (Catalog No: ETGA-200), EnzyChrom™ Free Fatty Acid Assay Kit (Catalog No: EFFA-100) and EnzyChrom™ Cholesterol Assay Kit (Catalog No: ECCH-100) from Bioassay Systems in USA, as per their manufacturers’ instructions, respectively.

### Lipid staining

BODIPY 493/503 (ThermoFisher), a fluorescence dye, was employed to visualize cellular neutral lipids following the protocols provided by the manufacturer. In brief, cells were fixed prior to incubation with the BODIPY 493/503. Subsequently, images were captured by using a confocal microscope.

### Evaluation of FAS and FAO

Detection assays for fatty acid synthesis (FAS) and oxidation (FAO) were performed by incubating hepatocellular carcinoma (HCC) cells with C^14^ citrate solution purchased from Moravek Biochemicals in Canada or H^3^ oleic acid solution obtained from Amersham Pharmacia Biotech in Italy for 16 h, respectively. The lipid phase or aqueous phase with C^14^ lipids or ^3^H_2_O was isolated, and the radioactivity was quantified using a L6500 liquid scintillation counter.

### Cell viability and colony formation assays

To assess cell proliferation, Cells seeded in 96-well plates (1 × 10^3^ cells/well) were incubated with MTS cell viability detection solution (Promega, G3581) at designated time points. The absorbance at a wavelength of 490 nm was finally recorded.

To assess the clonogenic ability, 500 HCC cells were plated in six-well plates and maintained 12 days. Subsequently, the colonies formed were fixed using 4% formaldehyde solution and stained with a 1% crystal violet solution. Finally, the colonies number were counted and compared.

### Lipid peroxidation

Lipid peroxidation levels were measured using C11-BODIPY 581/591 (Invitrogen, USA). Briefly, cells subjected to specified treatments were treated by C11-BODIPY 581/591 dye (5 μM) for 30 min at 37°C. Following incubation, 3 times washing were conducted using PBS solution, the results were measured using an Olympus BX51 fluorescence microscope (Olympus, Japan) at the excitation and emission maxima of 488 nm and 510 nm, respectively.

### Measurement of Fe^2+^ levels

To evaluate Fe^2+^ levels, HCC cells were treated by 5 µM FerroOrange (#36104, DOJINDO) for 25 minutes. Following incubation, 3 times washing were conducted using PBS solution. The results were visualized using a fluorescence microscope.

### Cell metastasis evaluation

The capabilities of cell migration and invasion were evaluated using the transwell assays in 24-well plates. In brief, cells (at a concentration of 1 × 10^5^ cells/well) were introduced into the chambers. After a 24-h incubation period for the migration assays or 48-h incubation for the invasion assays, the chambers were stained using crystal violet and the results were analyzed under a microscope.

### In vivo HCC proliferation and metastasis assays

BALB/c nude mice (4-week-old, male) in good health were obtained from Biological Sciences institute in Shanghai, China. Animal experiments were performed in accordance with the guidelines in the Institutional Animal Care and Use Committee of Xijing Hospital of the Air Force Medical University (No. 20230566). To reduce pain, suffering, and distress, personnel participates are adequately trained to possess proficient surgical skills to reduce procedural time. The animals were randomly separated into two groups (6 mice per group, 3 mice per cage) and maintained in a 12-h light/dark cycle with unrestricted standard pelleted diet and water under SPF (Specific Pathogen-Free) conditions. Heat-sterilized wood shavings were used for bedding. Animals were monitored daily and will be excluded if develop infections or tumor grows beyond 2 cm^3^. The experiment will be terminated if animals have severe pain, inability to eat or drink, or significant weight loss.

For in vivo growth ability analysis, 5 × 10^6^ cells were subcutaneously injected into the right flank of the nude mice. The body weight and behavioral changes were assessed every other day. Tumor length (L) and width (W) were measured weekly and their volume was calculated as: volume (cm^3^) = (*W*^2^ × *L*)/2. Four weeks post cells injection, the tumors were removed, weighed and then stained by immunohistochemistry.

To assess the in vivo metastasis ability, 3 × 10^6^ cells were intravenously injected into the nude mice through the tail vein. The body weight and behavioral changes were assessed every other day. At the end time point (6 weeks post cells injection), the lungs were collected and the metastatic nodes were examined by hematoxylin-eosin (HE) staining.

### Bioinformatics analysis

C12ORF49 expression in HCC was analyzed using the UALCAN platform (http://ualcan.path.uab.edu/), a comprehensive web resource for analyzing cancer transcriptomics data from The Cancer Genome Atlas (TCGA) database [30]. Briefly, the expression of C12ORF49 was compared between HCC and normal liver tissues using box plots. Statistical significance (p-value) of differential expression was assessed using the built-in tools in UALCAN. In addition, Kaplan–Meier survival analysis was conducted to evaluate the relationship between C12ORF49 expression and patient survival using the built-in tools in UALCAN.

Analysis of C12ORF49 expression in Pan-cancer was conducted using Sangerbox 3.0 (http://www.sangerbox.com/), a versatile bioinformatics tool for integrative analysis of multi-omics data across various cancer types [31]. C12ORF49 expression was analyzed across multiple cancer datasets available in the TCGA database. Gene expression levels were visualized using box plots and scatter plots generated by Sangerbox 3.0. The correlation between C12ORF49 expression and patient hazard ratio across different types of cancer was analyzed using the Cox regression model in Sangerbox 3.0.

### Construction of the sorafenib-resistant HCC cells

Sorafenib-resistant HCC cells were established in HLF and HLE cells by adding step-wise elevating Sorafenib concentration (2 µM, two weeks; 4 µM, 2 weeks; 6 µM, 2 weeks; 8 µM, 2 weeks; 10 µM, 2 weeks) into the medium during repeated passages.

### TUNEL staining assay

To assess apoptosis in xenograft tissues, a terminal deoxynucleotidyl transferase-mediated dUTP nick-end labeling (TUNEL) assay kit from Roche (Catalog No: 11684795910) was used, following the manufacturer’s instructions. DAPI was used for nuclear staining. The staining images were captured using an Olympus BX51 fluorescence microscope (Olympus, Japan) and the percentages of apoptotic cells (TUNEL-positive nuclei) were compared between shCtrl and shC12ORF49 groups.

### Statistical analysis

Data are reported as mean ± SEM, with statistical analysis performed using GraphPad Prism version 6.0. The student’s t-test or the Mann–Whitney *U* test was used to compare individual data between two groups when the variance was homogeneous or non-homogeneous. The one-way ANOVA test was employed for comparisons of individual data between more than two experimental groups. Patient survival was compared using Kaplan–Meier method. The relationship between C12ORF49 and patients’ clinicopathological variables was analyzed by *χ*^2^ tests. A *p*-value less than 0.05 was indicated as statistical significance (*).

## Supplementary information

Supplementary figures and tables

Full and uncropped western blots

## Data Availability

All data supporting the findings of this study are available from the corresponding author upon reasonable request.
