# Supplementary material for: C12ORF49 inhibits ferroptosis in hepatocellular carcinoma cells via reprogramming SREBP1/SCD1-mediated lipid metabolism
Source: Cell Death Discov. 2025 Apr 16;11:178. doi: 10.1038/s41420-025-02480-2 (PMC12003882; doi:10.1038/s41420-025-02480-2)
Supplement: Supplementary file 1 — Supplementary figures and tables [file 41420_2025_2480_MOESM1_ESM.docx]

**Supplemental information**

**C12ORF49 inhibits ferroptosis in hepatocellular carcinoma cells via reprogramming SREBP1/SCD1-mediated lipid metabolism**

**Supplemental figures**

**Figure S1.** **Pan-cancer analyses of the expressions of C12ORF49 (A) and the association between C12ORF49 level and patients’ survival (B) using the online Sangerbox 3.0 database.**


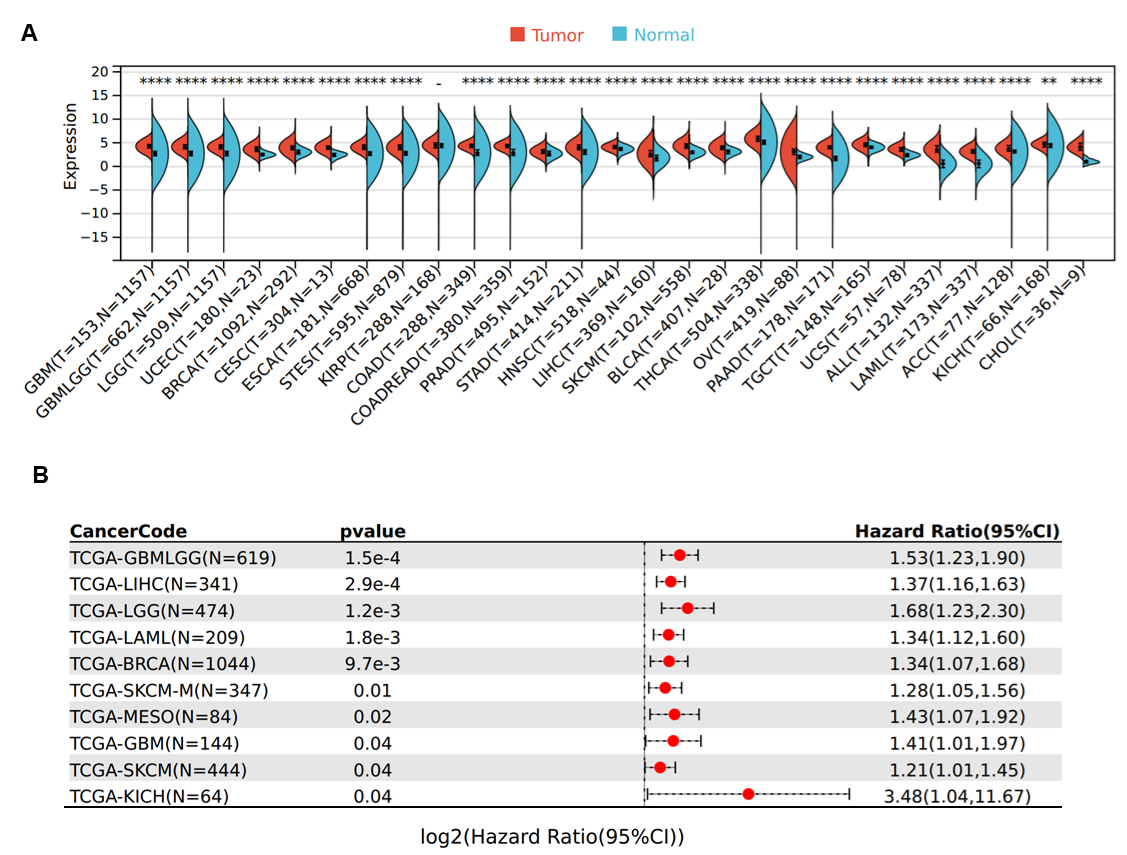


**Figure S2.** **C12ORF49 knockdown had no significant effect on the migration and invasion of HCC cells. (A and B)** The impact of C12ORF49 knockdown on cell metastasis was tested by wound healing (A) and transwell invasion (B) assays in HLF and HLE cells. **(C and D)** The impact of C12ORF49 knockdown on epithelial-mesenchymal-transition (EMT) was tested by qRT-PCR (C) and western blot (D) assays by evaluating the expressions of epithelial and mesenchymal markers in HLF and HLE cells.

**
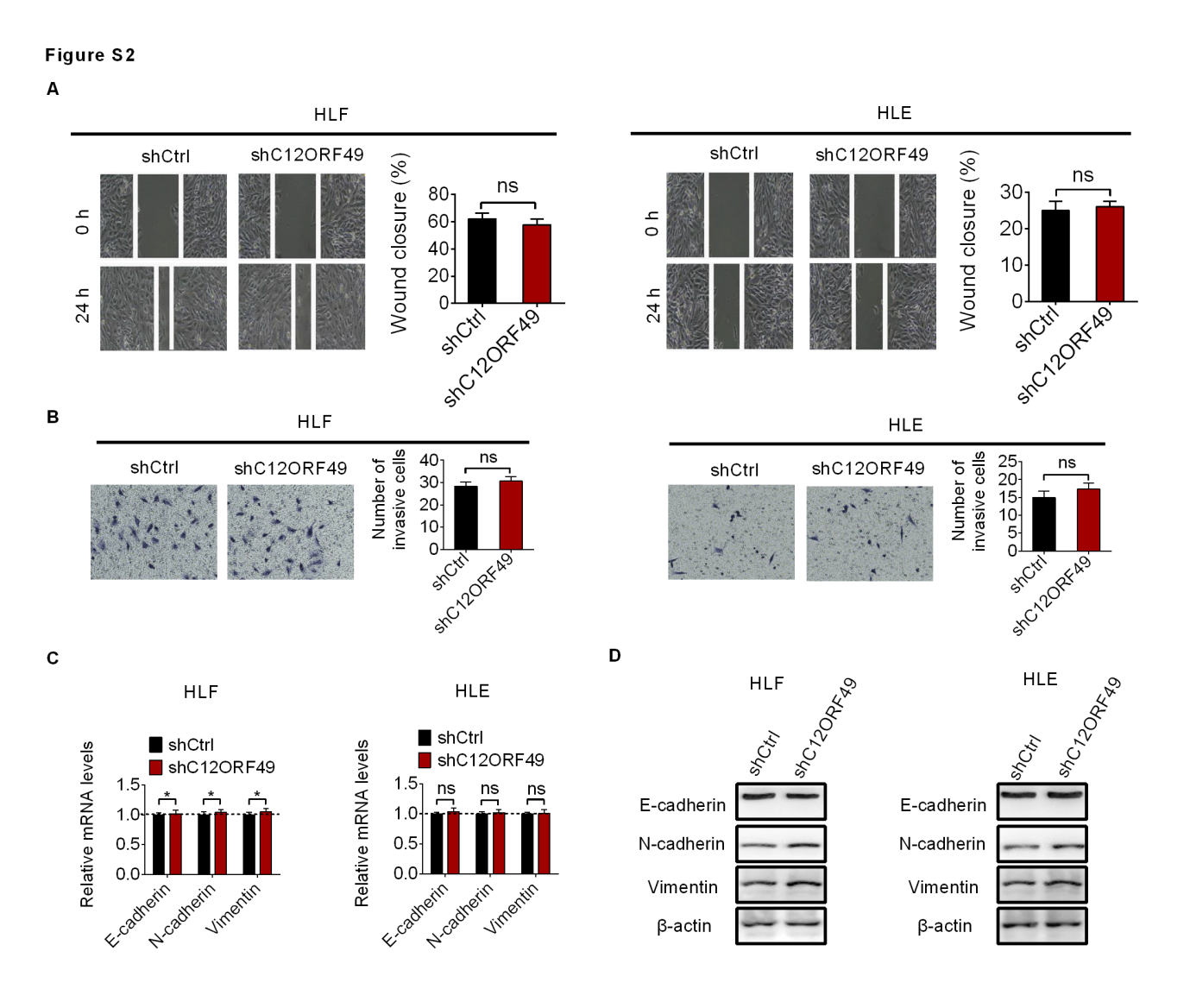
**

**Figure S3. (A)**The effect of C12ORF49 on the expressions of fatty acid synthesis enzymes in HCC cells was assessed using qRT-PCR assay. **(B)** Representative IHC staining images of C12ORF49, SREBP1 and SCD1 in HCC tissues.

**
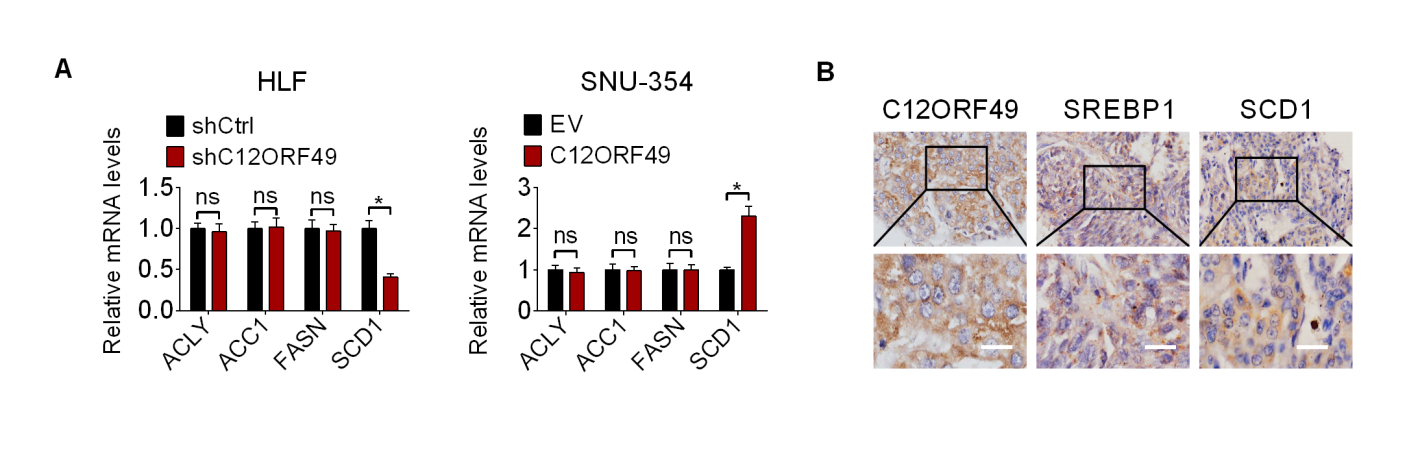
**

**Supplemental Tables**

**Table S1.** Sequence of primers used for qRT-PCR analysis

| **1. Primers used in q-PCR analysis** | | |  |
| --- | --- | --- | --- |
| *C12ORF49* | forward primer | ACTTGGGCAATAGCAGTCGTC | |
|  | reverse primer | GCAAACGTAGCCGAGTTCAT | |
| *E-cadherin* | forward primer | ATTTTTCCCTCGACACCCGAT | |
|  | reverse primer | TCCCAGGCGTAGACCAAGA | |
| *Vimentin* | forward primer | GCAAAGATTCCACTTTGCGT | |
|  | reverse primer | GAAATTGCAGGAGGAGATGC | |
| *N-cadherin* | forward primer | TCAGGCTGTGGACATAGAAACC | |
|  | reverse primer | GCTGTAAACGACTCTGGCACT | |
| *SREBP1* | forward primer | ACAGTGACTTCCCTGGCCTAT | |
|  | reverse primer | GCATGGACGGGTACATCTTCAA | |
| *SREBP2* | forward primer | TGGCTTCTCTCCCTACTCCA | |
|  | reverse primer | GAGAGGCACAGGAAGGTGAG | |
| *ACLY* | forward primer | TCGGCCAAGGCAATTTCAGAG | |
|  | reverse primer | CGAGCATACTTGAACCGATTCT | |
| *FASN* | forward primer | AAGGACCTGTCTAGGTTTGATGC | |
|  | reverse primer | TGGCTTCATAGGTGACTTCCA | |
| *ACC1* | forward primer | ATGTCTGGCTTGCACCTAGTA | |
|  | reverse primer | CCCCAAAGCGAGTAACAAATTCT | |
| *SCD1* | forward primer | AGAATGGAGGAGATAAGT | |
|  | reverse primer | TAGCAGAGACATAAGGAT | |
| *β-actin* | forward primer | ATCAAGATCATTGCTCCTCCTGAG | |
|  | reverse primer | CTGCTTGCTGATCCACATCTG | |

**Table S2.** Primary antibodies used for western blotting and IHC staining assays.

| **Antibody** | **Company (Cat. No.)** | **Working dilutions** |
| --- | --- | --- |
| C12ORF49 | Thermo Fisher (PA5-55459) | WB: 1/800; IHC:1/150 |
| E-cadherin | Proteintech (20874-1-AP) | WB: 1/1000 |
| Vimentin | Proteintech (10366-1-AP) | WB: 1/1000 |
| N-cadherin | Proteintech (22018-1-AP) | WB: 1/1000 |
| SREBP1 | Proteintech (66875-1-Ig) | WB: 1/1000 |
| SREBP2 | Proteintech (28212-1-AP) | WB: 1/1000 |
| SCD1 | Proteintech (23393-1-AP) | WB: 1/1000 |
| β-actin | Proteintech (20536-1-AP) | WB: 1/1000 |

**Table 3.** **Correlation between the expressions of C12ORF49 and clinicopathologic features of 238 HCC patients.**

| Variables | No. of cases (%) | C12ORF49 expression | | *P* value |
| --- | --- | --- | --- | --- |
|  |  | Low | High |  |
| All | 238 (100%) | 119 | 119 |  |
| Age |  |  |  |  |
| <=55 | 91 (38.2%) | 43 | 48 | 0.594 |
| >55 | 147 (61.8%) | 76 | 71 |  |
| Gender |  |  |  |  |
| Female | 42 (17.6%) | 23 | 19 | 0.610 |
| Male | 196 (82.4%) | 96 | 100 |  |
| HBV |  |  |  |  |
| Negative | 28 (11.8%) | 11 | 17 | 0.314 |
| Positive | 210 (88.2%) | 108 | 102 |  |
| Alpha-fetoprotein  (ug/ml) |  |  |  |  |
| <=200 | 129 (54.2%) | 68 | 61 | 0.435 |
| >200 | 109 (45.8%) | 51 | 58 |  |
| Maximum diameter of lesion |  |  |  |  |
| <=5 | 122 (51.3%) | 70 | 52 | **0.027** |
| >5 | 116 (48.7%) | 49 | 67 |  |
| PVTT |  |  |  |  |
| No | 196 (82.4%) | 106 | 90 | **0.010** |
| Yes | 42 (17.6 %) | 13 | 29 |  |
| TNM stage |  |  |  |  |
| I+ II | 185 (77.7%) | 97 | 88 | 0.212 |
| III+ IV | 53 (22.3%) | 22 | 31 |  |
| Differentiation grade |  |  |  |  |
| I+ II | 77 (32.4%) | 43 | 34 | 0.268 |
| III | 161 (77.6%) | 76 | 85 |  |

**Abbreviations**: PVTT, portal vein tumor thrombosis; TNM, tumor-node-metastasis; TNM stage I (T1N0M0), II (T2N0M0), III (T3N0M0, T4N0M0, TxN1M0) and stage IV (TxNxM1).
