# Supplementary material for: C12ORF49 inhibits ferroptosis in hepatocellular carcinoma cells via reprogramming SREBP1/SCD1-mediated lipid metabolism
Source: Cell Death Discov. 2025 Apr 16;11:178. doi: 10.1038/s41420-025-02480-2 (PMC12003882; doi:10.1038/s41420-025-02480-2)
Supplement: Supplementary file 2 — Full and uncropped western blots [file 41420_2025_2480_MOESM2_ESM.pdf]

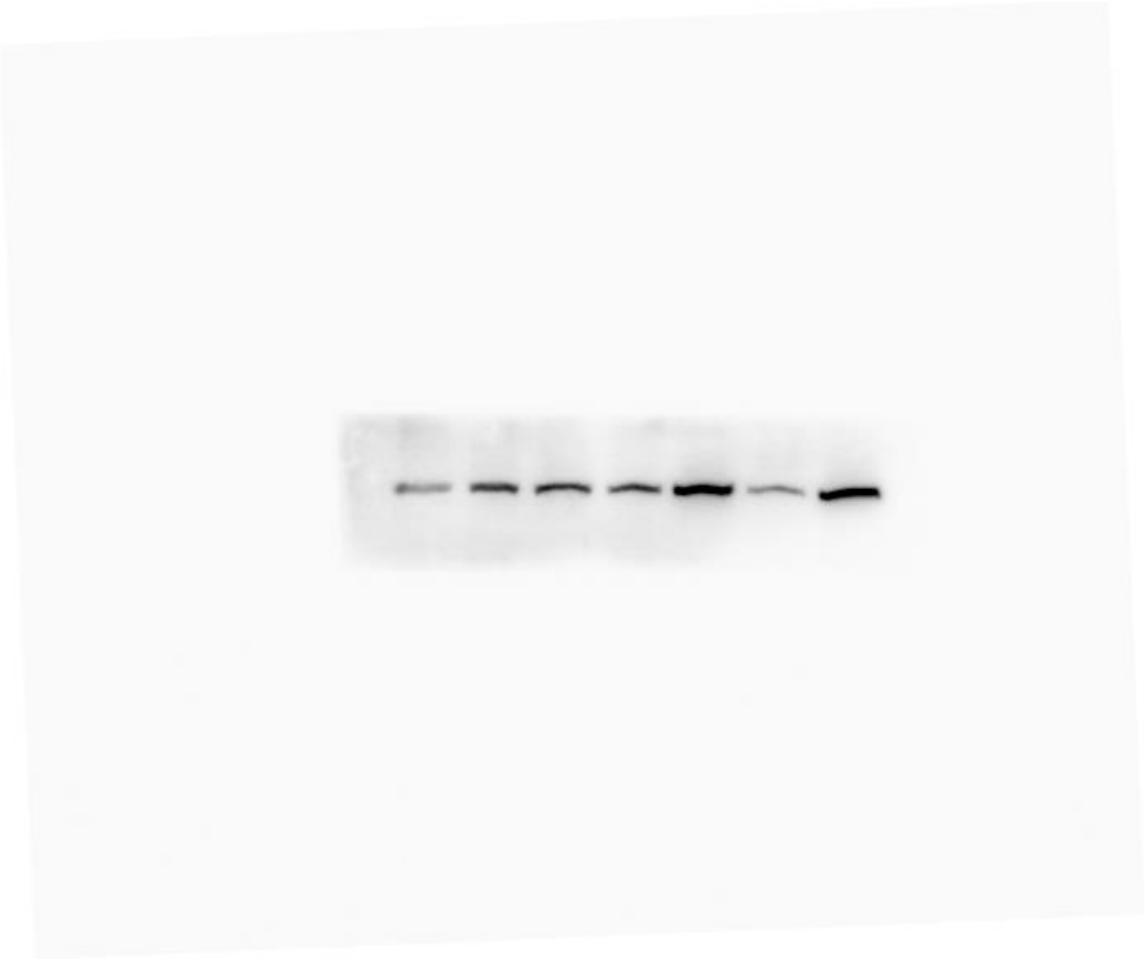

Fig 1B (IB: C12ORF49);

Line 1-HL-7702, Line 2-Huh-7, Line 3-SNU-368, Line 4-SNU-354,

Line 5-HLF, Line 6-SNU-739, Line 7-HLE

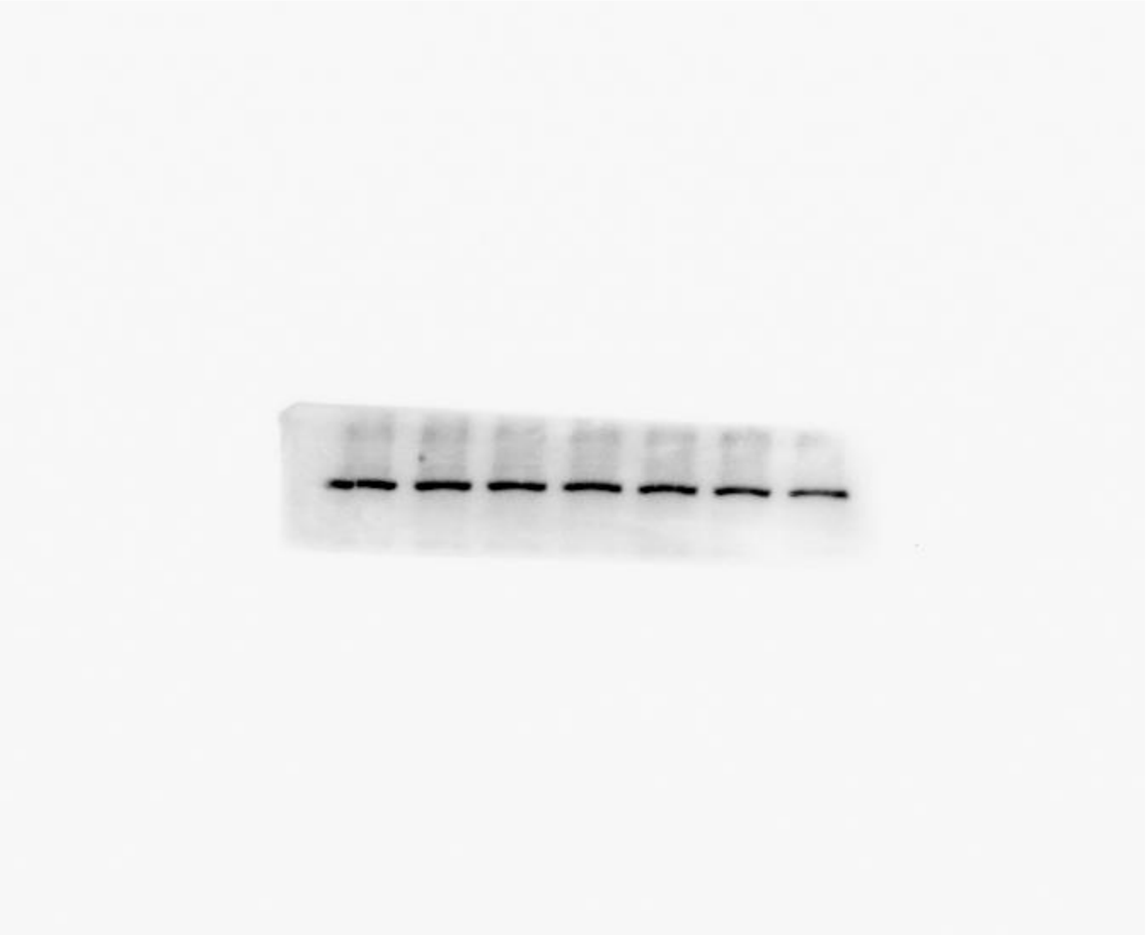

Fig 1B (IB: β-actin);

Line 1-HL-7702, Line 2-Huh-7, Line 3-SNU-368, Line 4-SNU-354,

Line 5-HLF, Line 6-SNU-739, Line 7-HLE

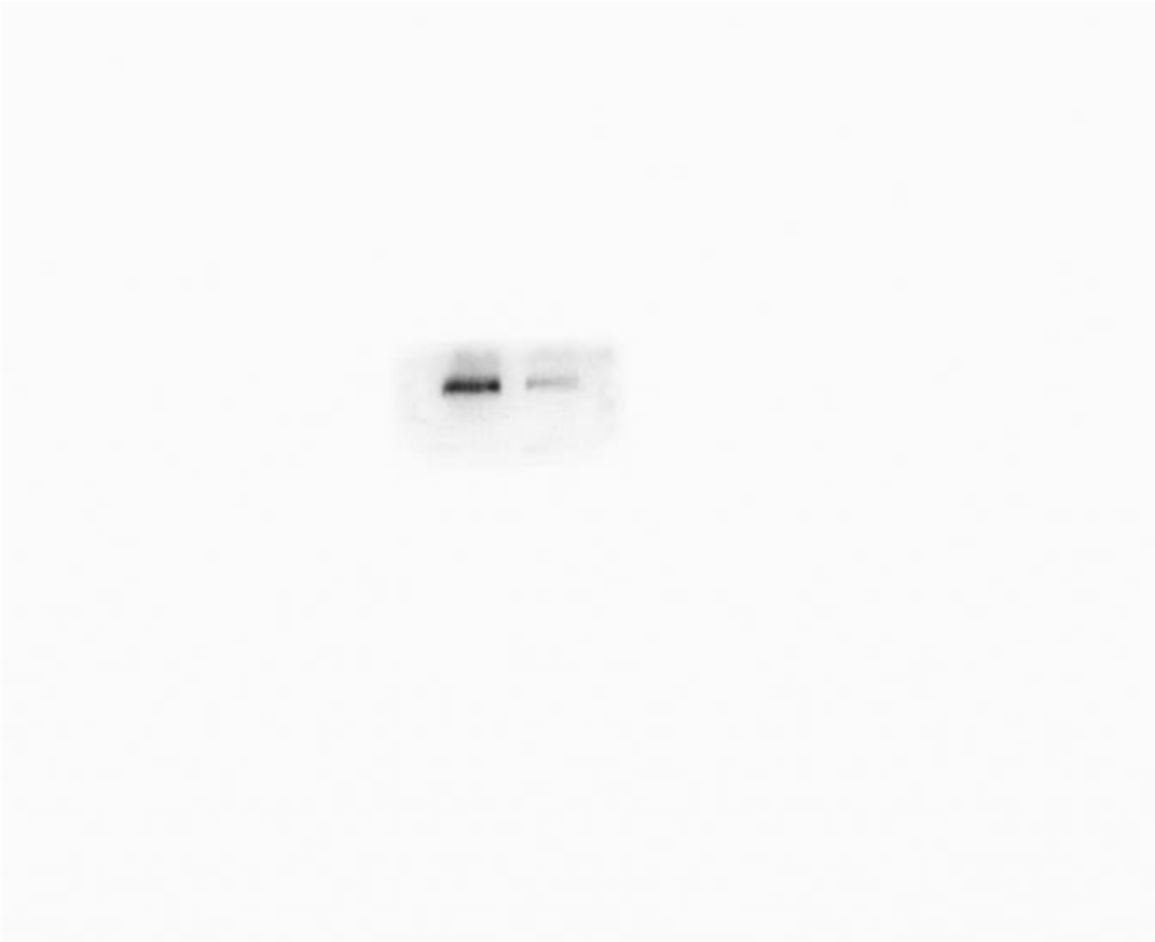

Fig 2B (IB: C12ORF49 in HLF cells);

Line 1-shCtrl, Line2-shC12ORF49.

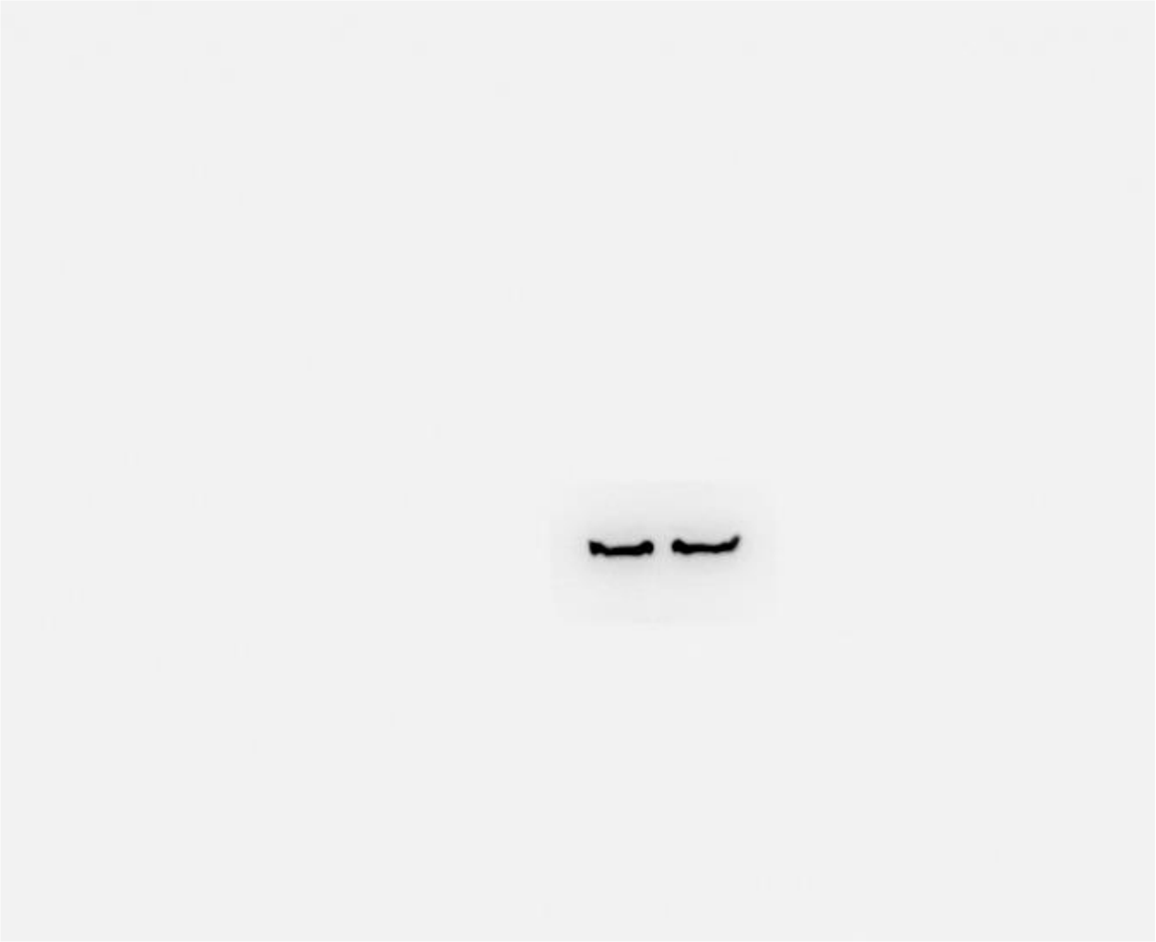

Fig 2B (IB: β-actin in HLF cells);

Line 1-shCtrl, Line2-shC12ORF49.

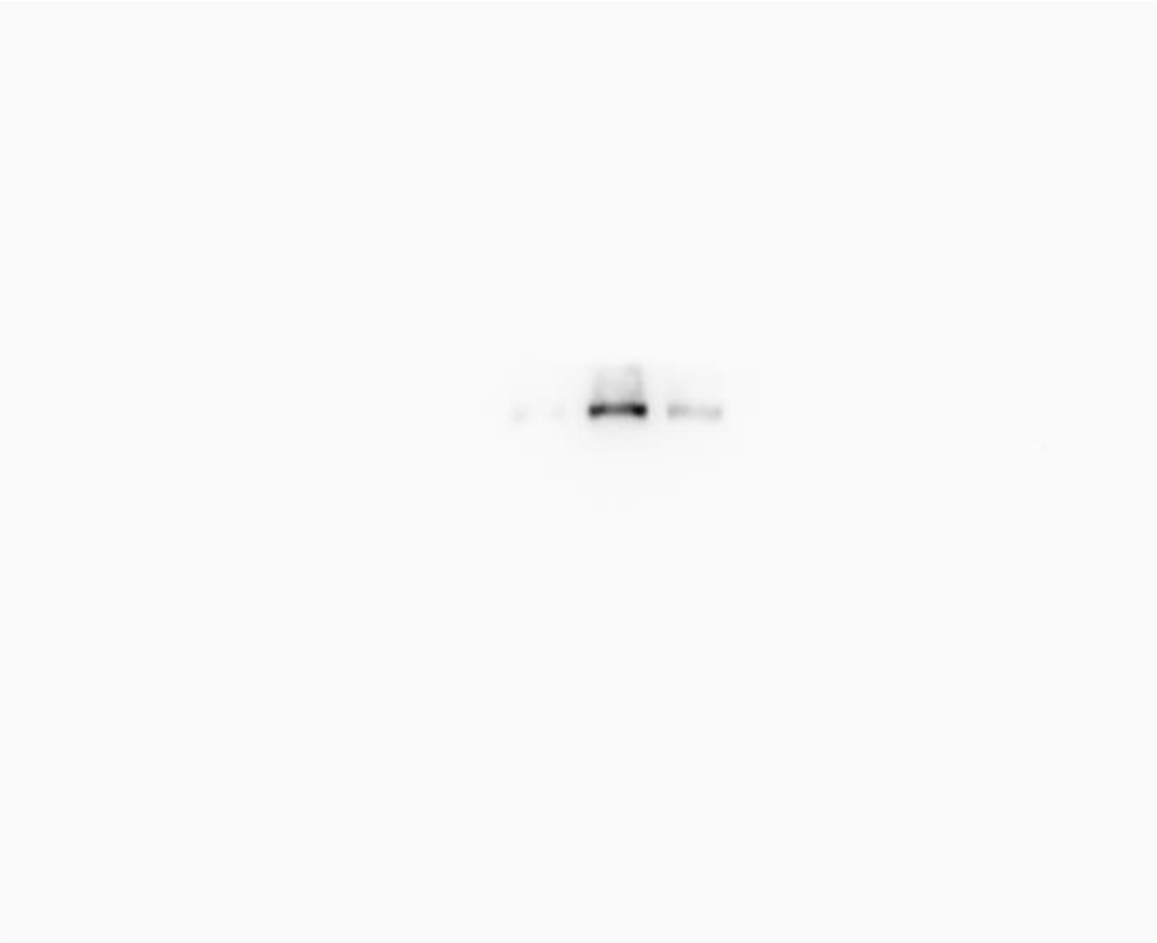

Fig 2B (IB: C12ORF49 in HLE cells);

Line 1-shCtrl, Line 2-shC12ORF49.

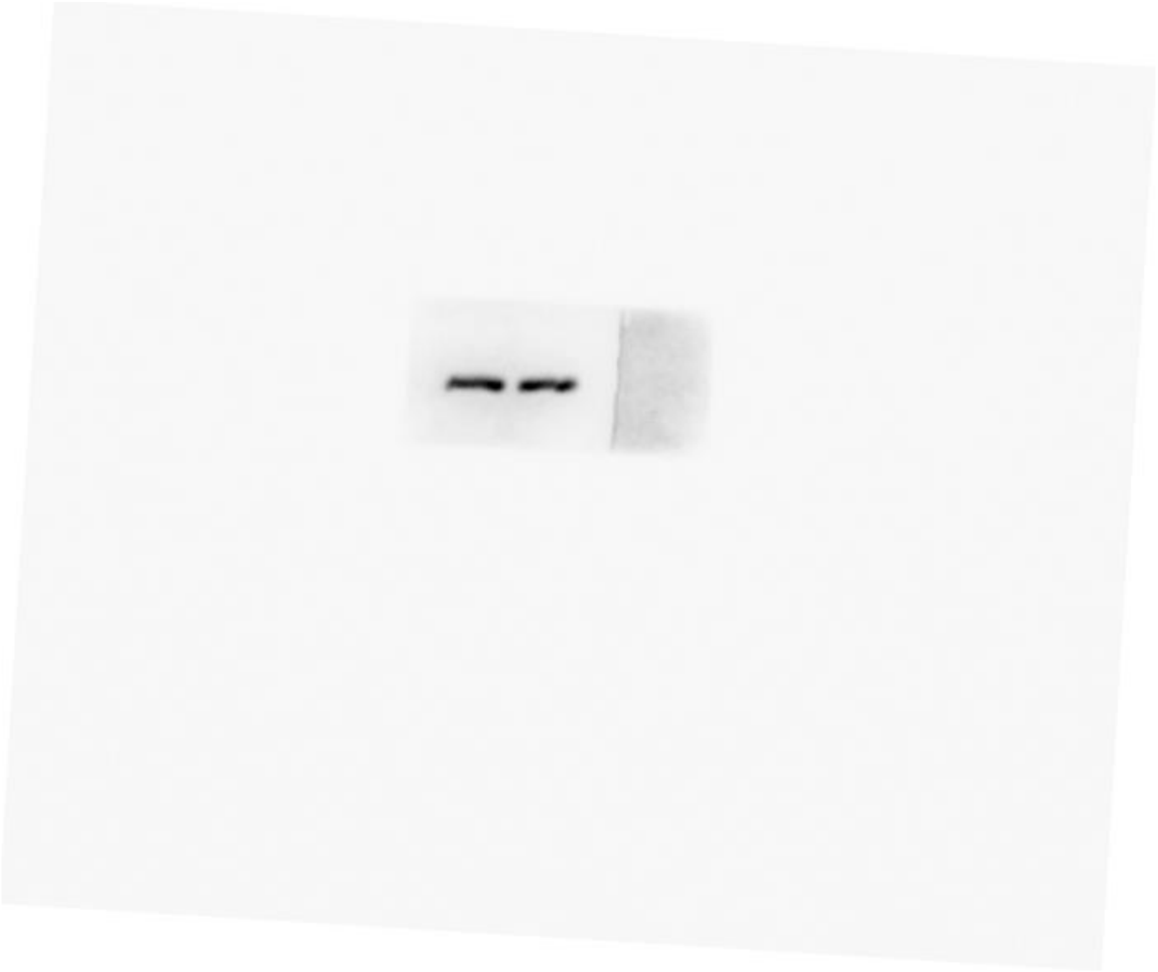

Fig 2B (IB: β-actin in HLE cells);

Line 1-shCtrl, Line 2-shC12ORF49.

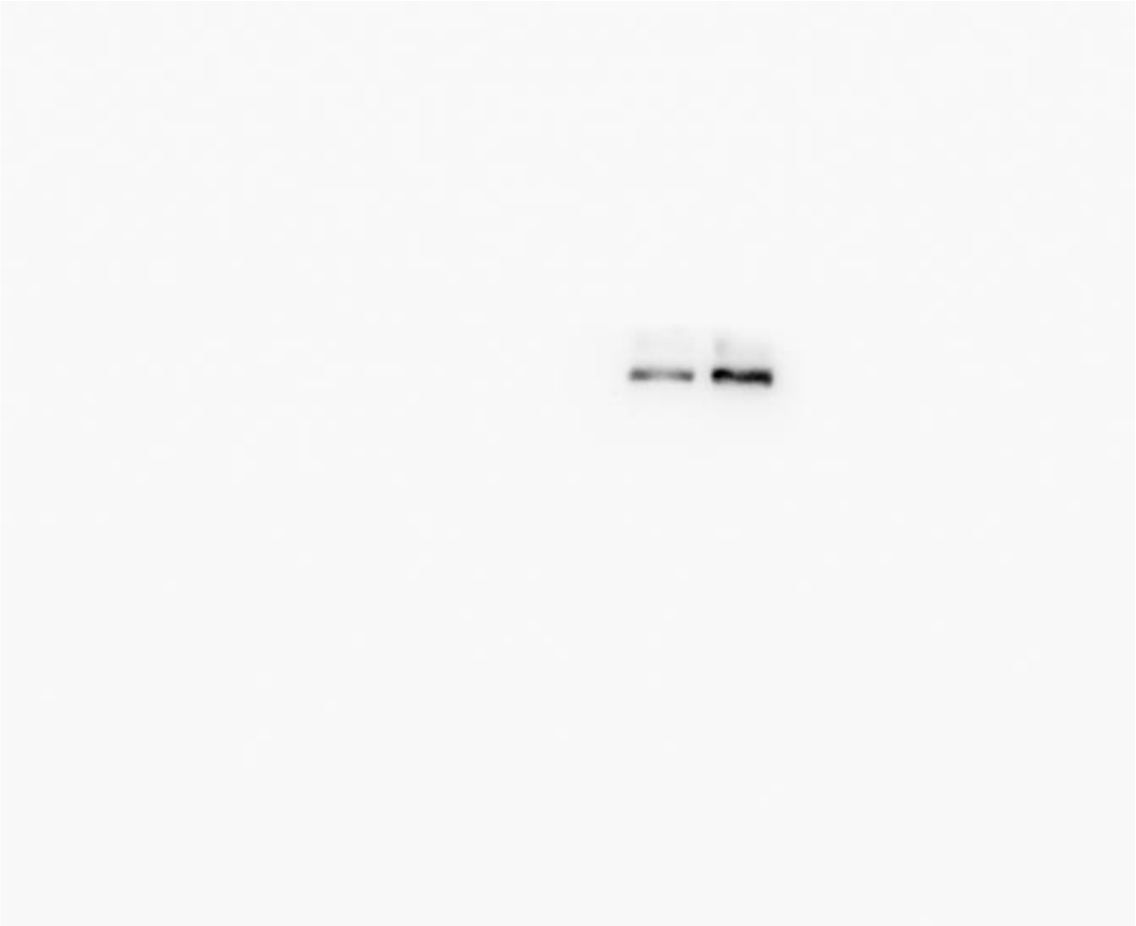

Fig 4B (IB: C12ORF49 in SNU-354 cells);

Line 1-EV, Line 2-C12ORF49

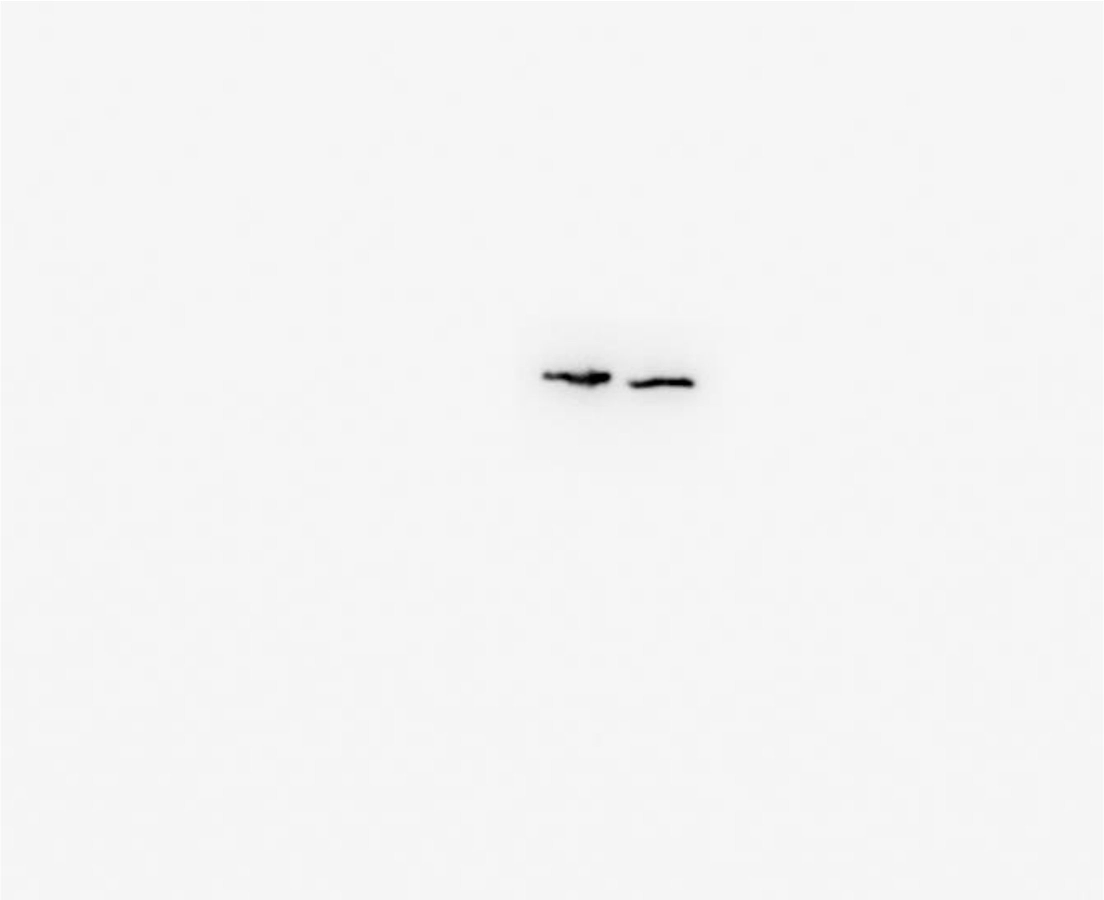

Fig 4B (IB: β-actin in SNU-354 cells);

Line 1-EV, Line 2-C12ORF49

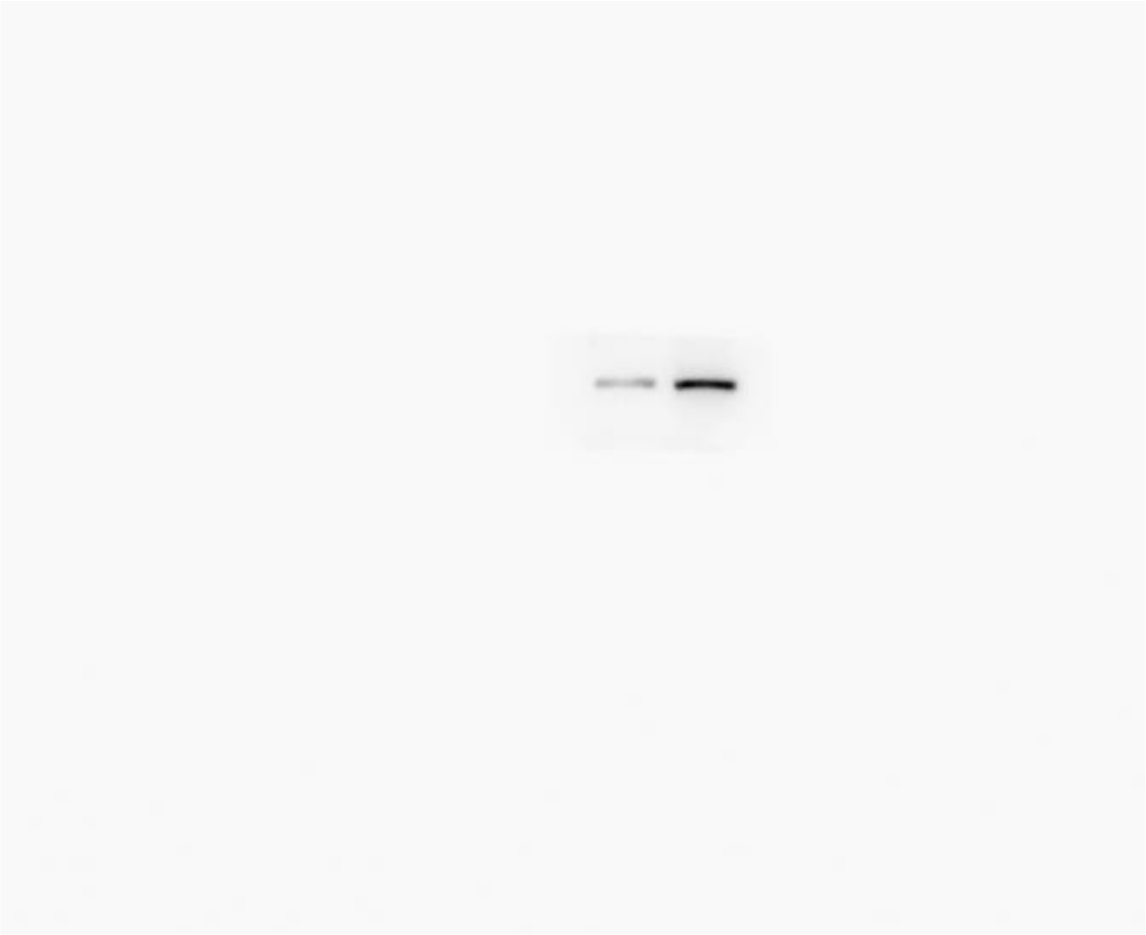

Fig 4B (IB: C12ORF49 in SNU-739 cells);

Line 1-EV, Line 2-C12ORF49

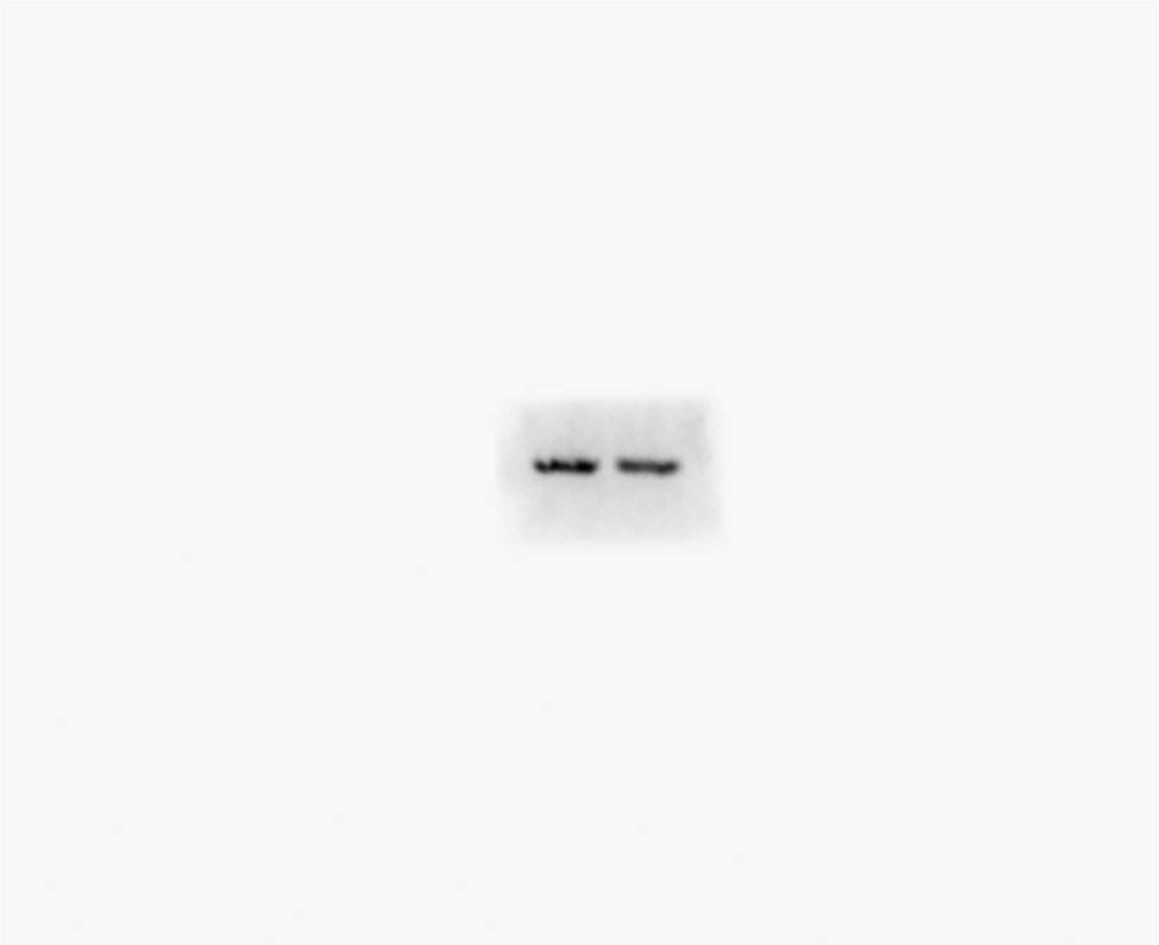

Fig 4B (IB: β-actin in SNU-739 cells);

Line 1-EV, Line 2-C12ORF49

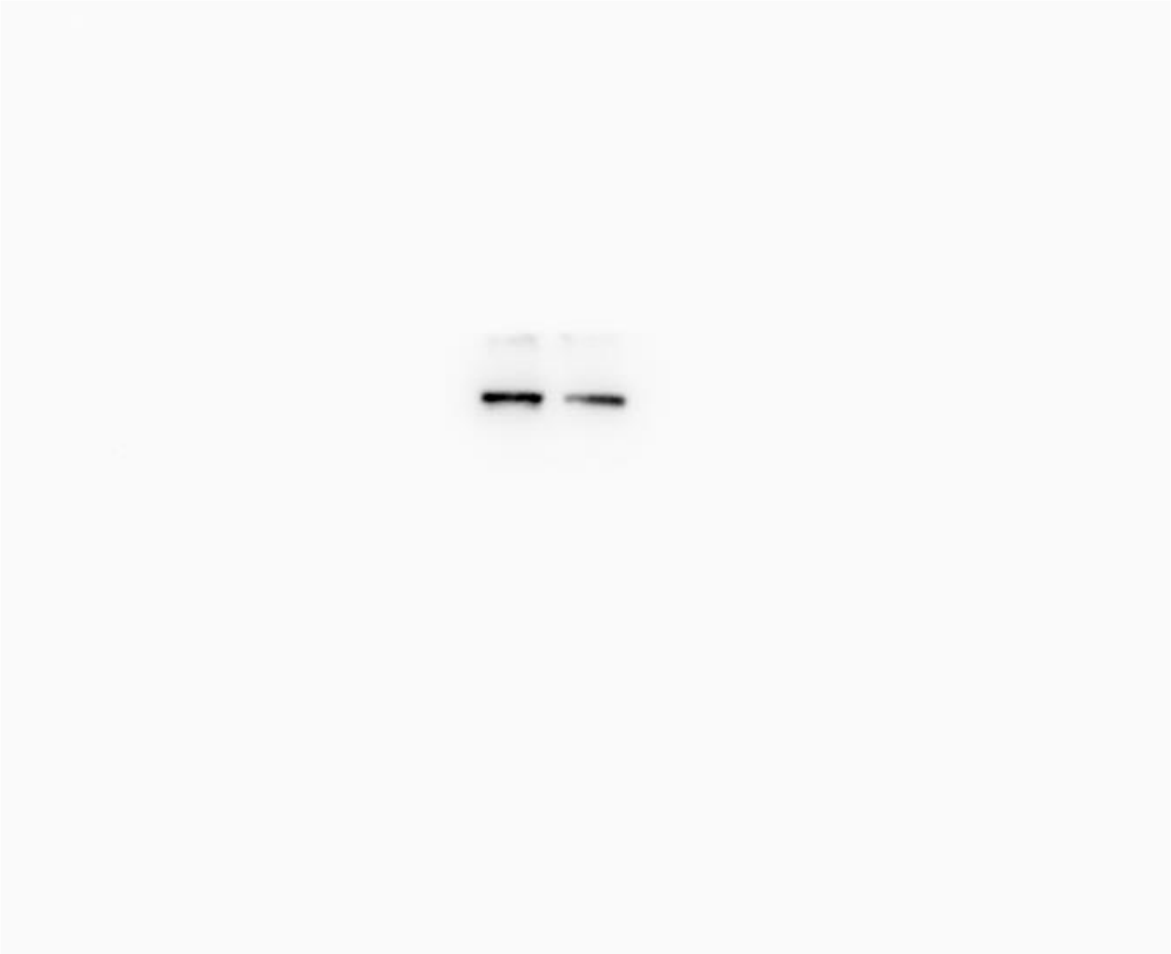

Fig 6F (IB: SREBP1 in HLF cells);

Line 1-shCtrl, Line 2-shC12ORF49.

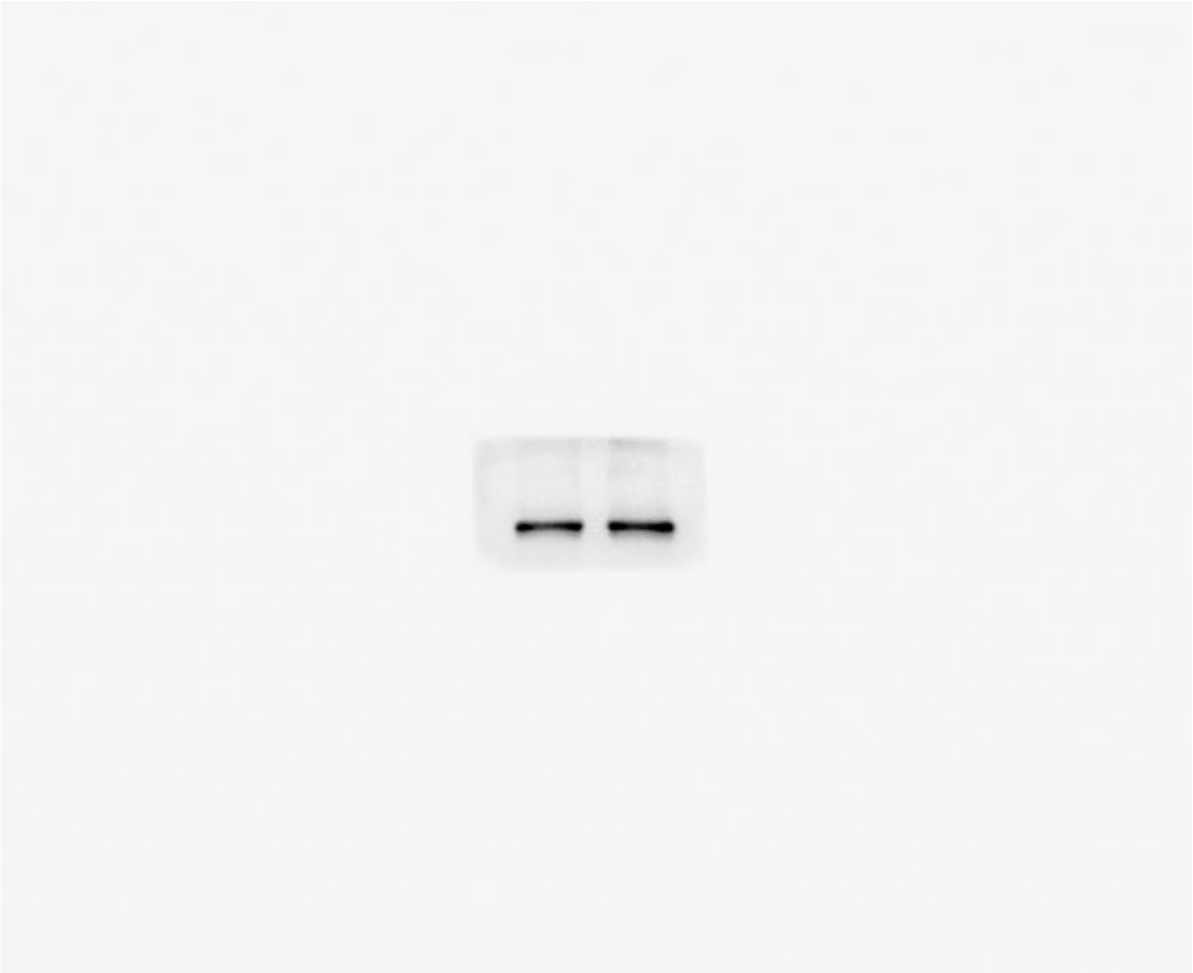

Fig 6F (IB: SREBP2 in HLF cells);

Line 1-shCtrl, Line 2-shC12ORF49.

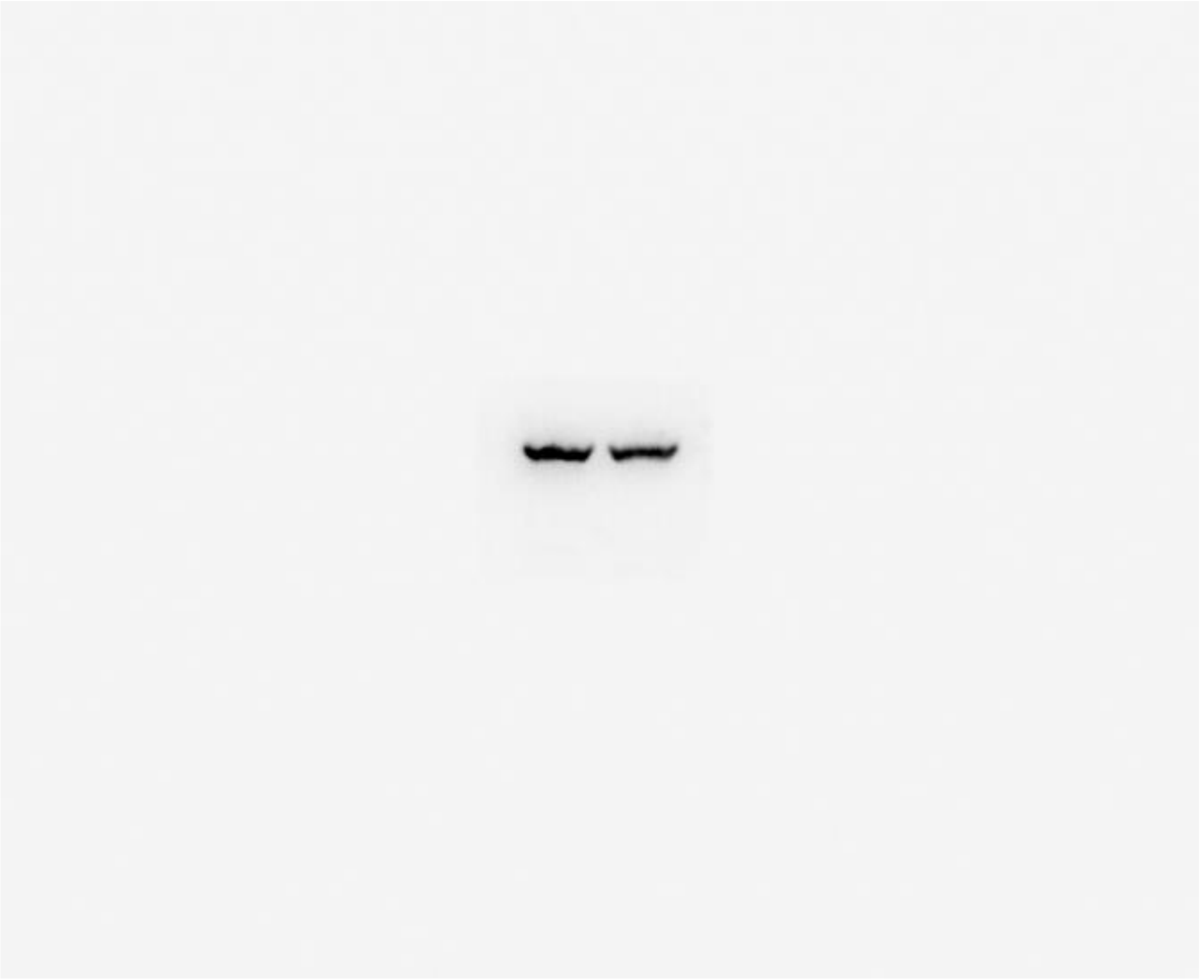

Fig 6F (IB: β-actin in HLF cells);

Line 1-shCtrl, Line 2-shC12ORF49.

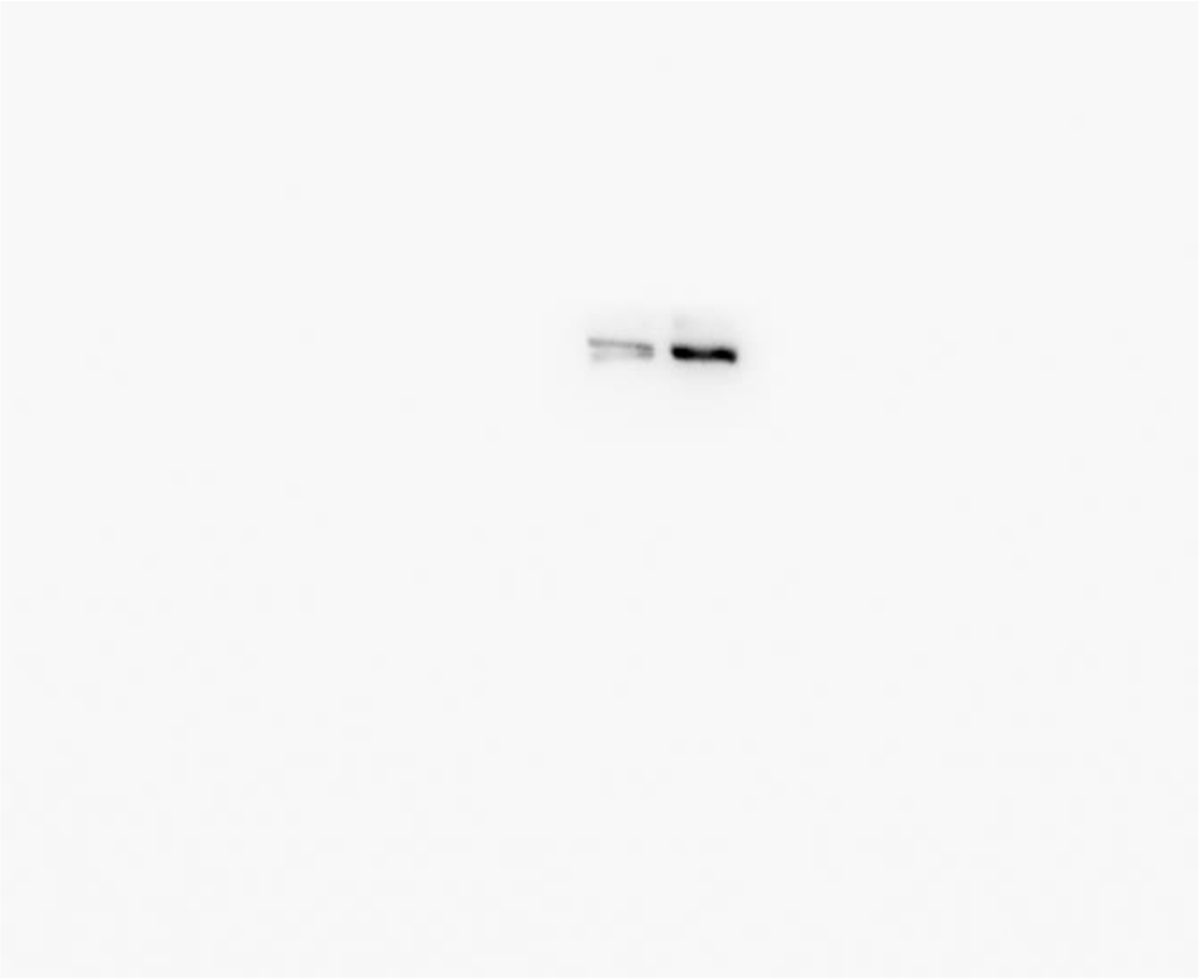

Fig 6F (IB: SREBP1 in SNU-354 cells);

Line 1-EV, Line 2-C12ORF49.

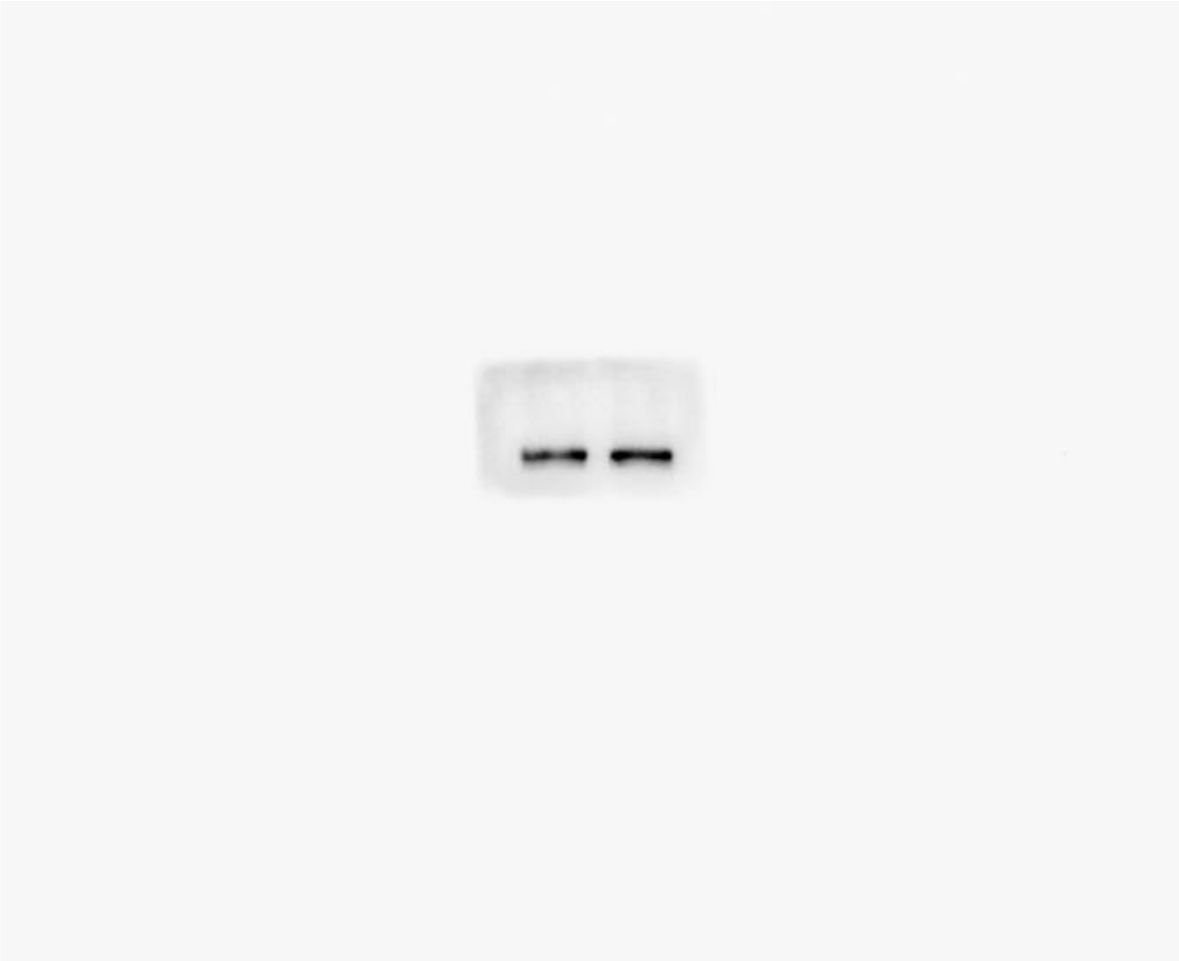

Fig 6F (IB: SREBP2 in SNU-354 cells);

Line 1-EV, Line 2-C12ORF49.

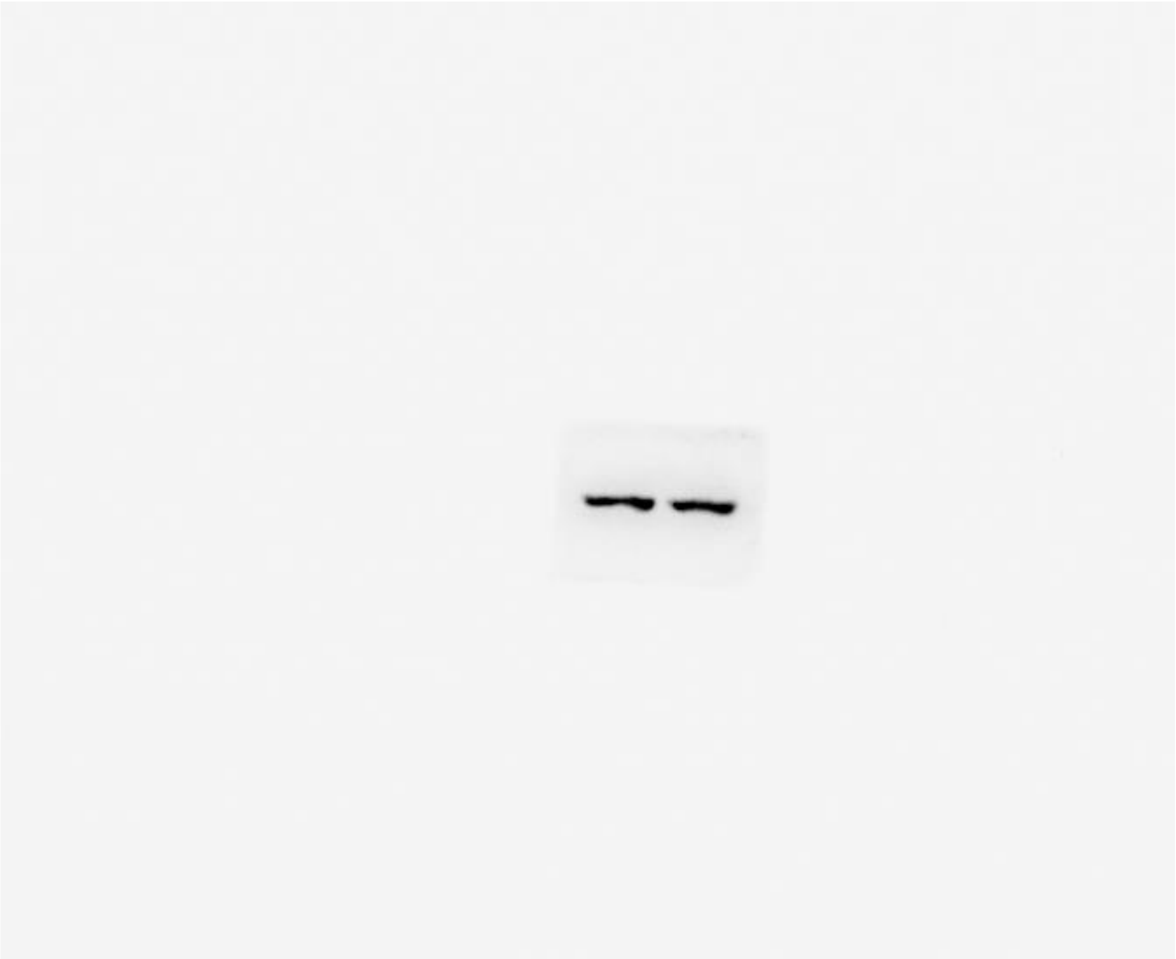

Fig 6F (IB: β-actin in SNU-354 cells);

Line 1-EV, Line 2-C12ORF49.

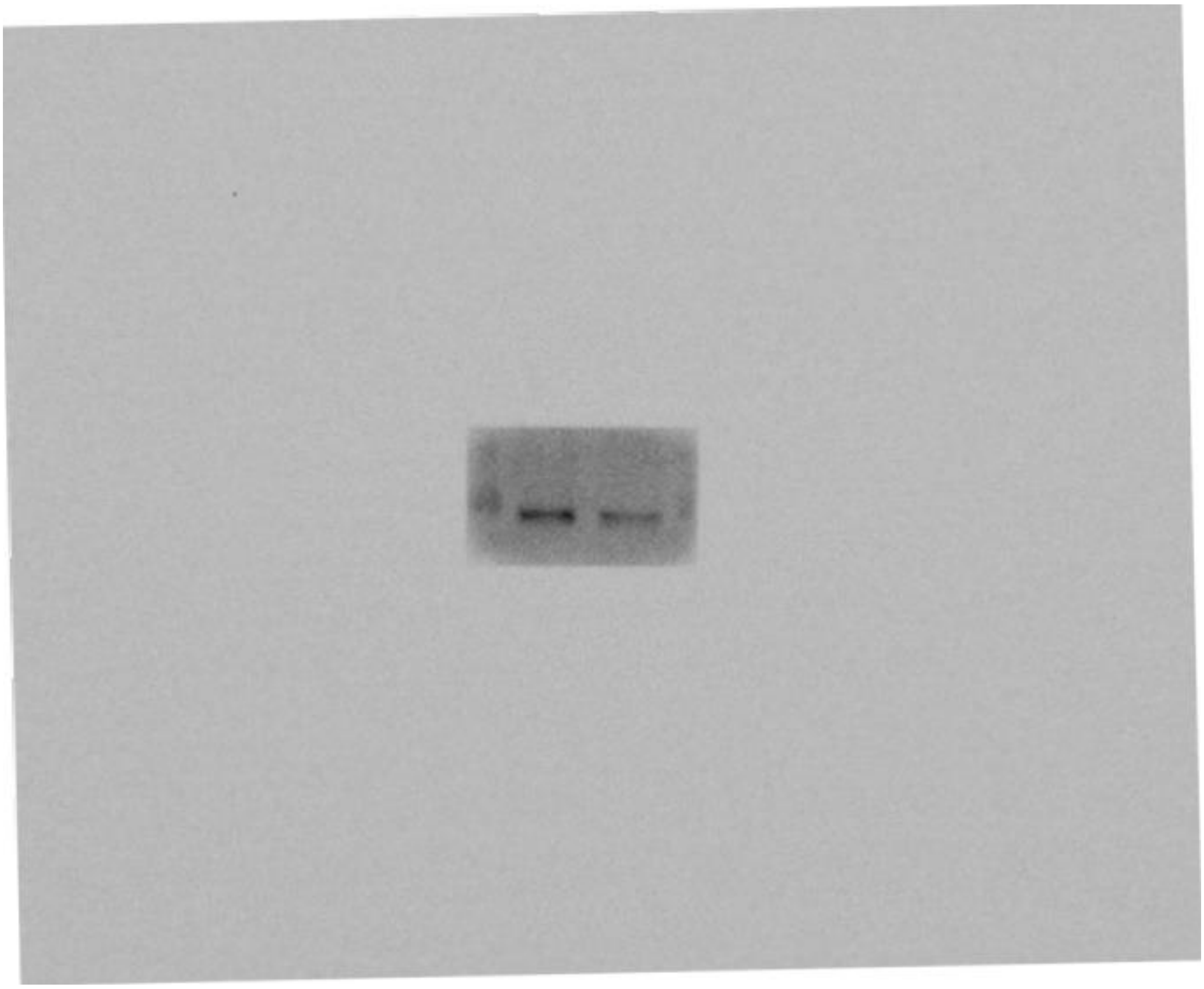

Fig 6G (IB: Nuclear SREBP1 in HLF cells);

Line 1-EV, Line 2-C12ORF49.

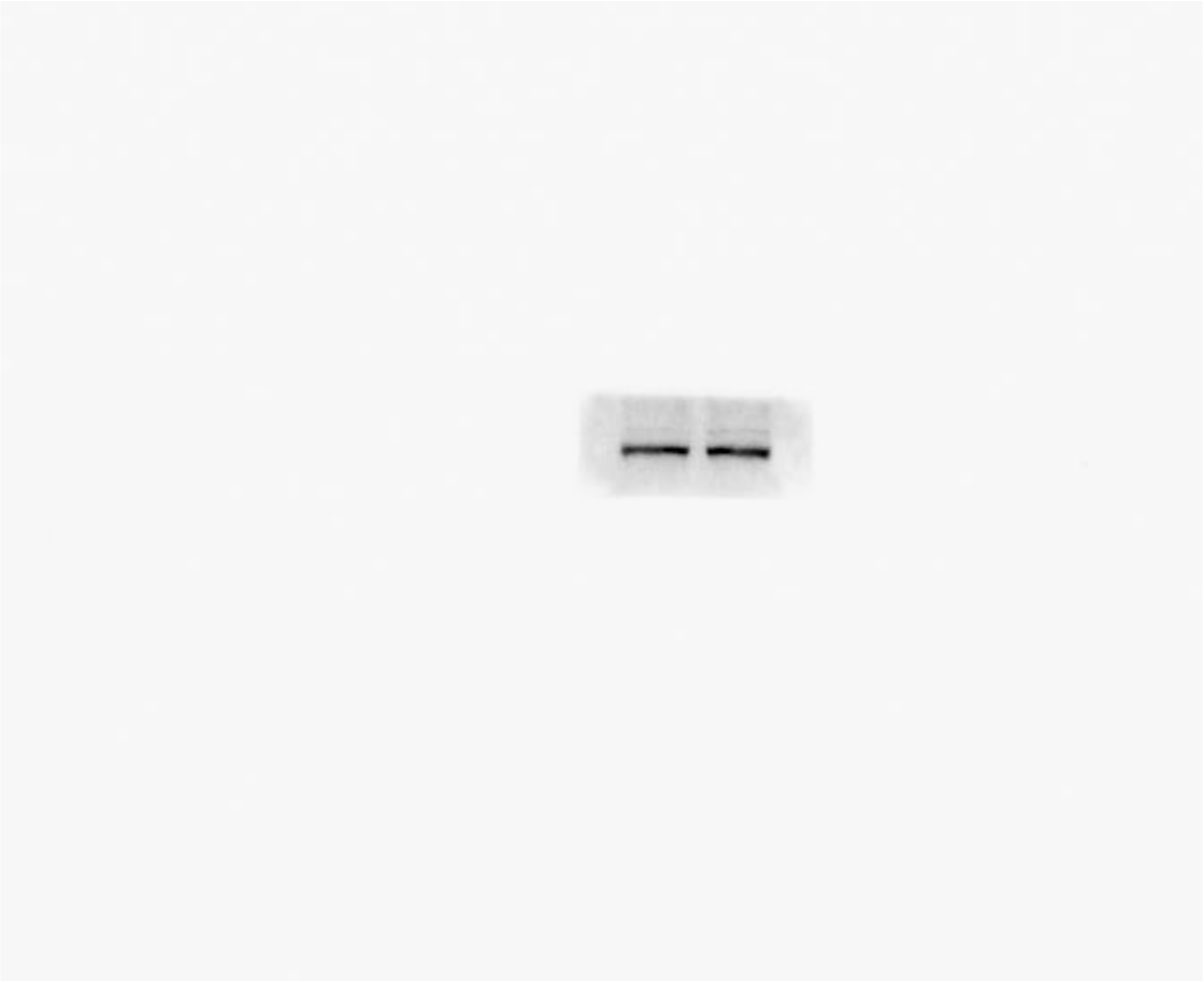

Fig 6G (IB: Lamin B1 in HLF cells);

Line 1-shCtrl, Line 2-shC12ORF49.

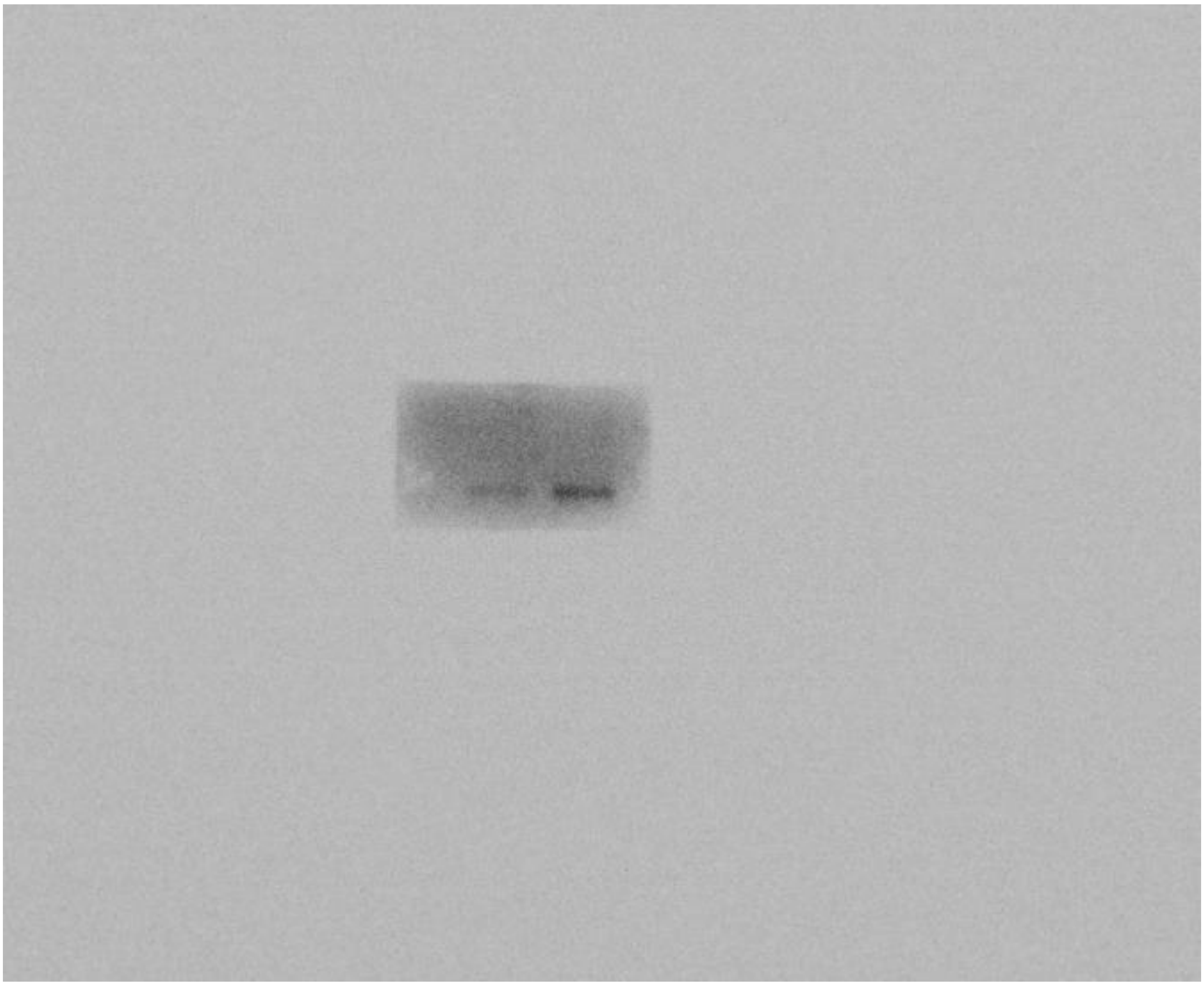

Fig 6G (IB: Nuclear SREBP1 in SNU-354 cells);

Line 1-EV, Line 2-C12ORF49.

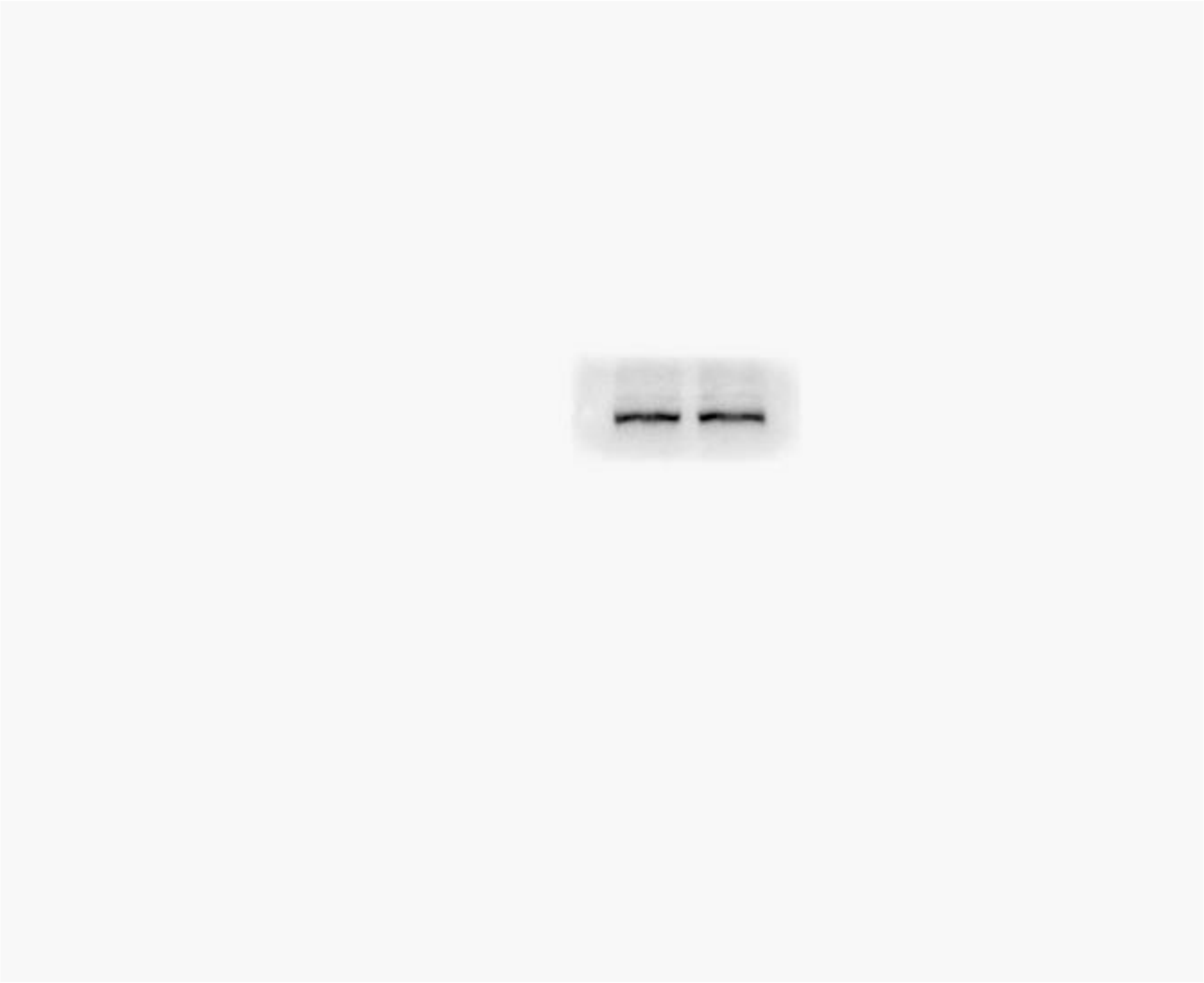

Fig 6G (IB: Lamin B1 in SNU-354 cells);

Line 1-EV, Line 2-C12ORF49.

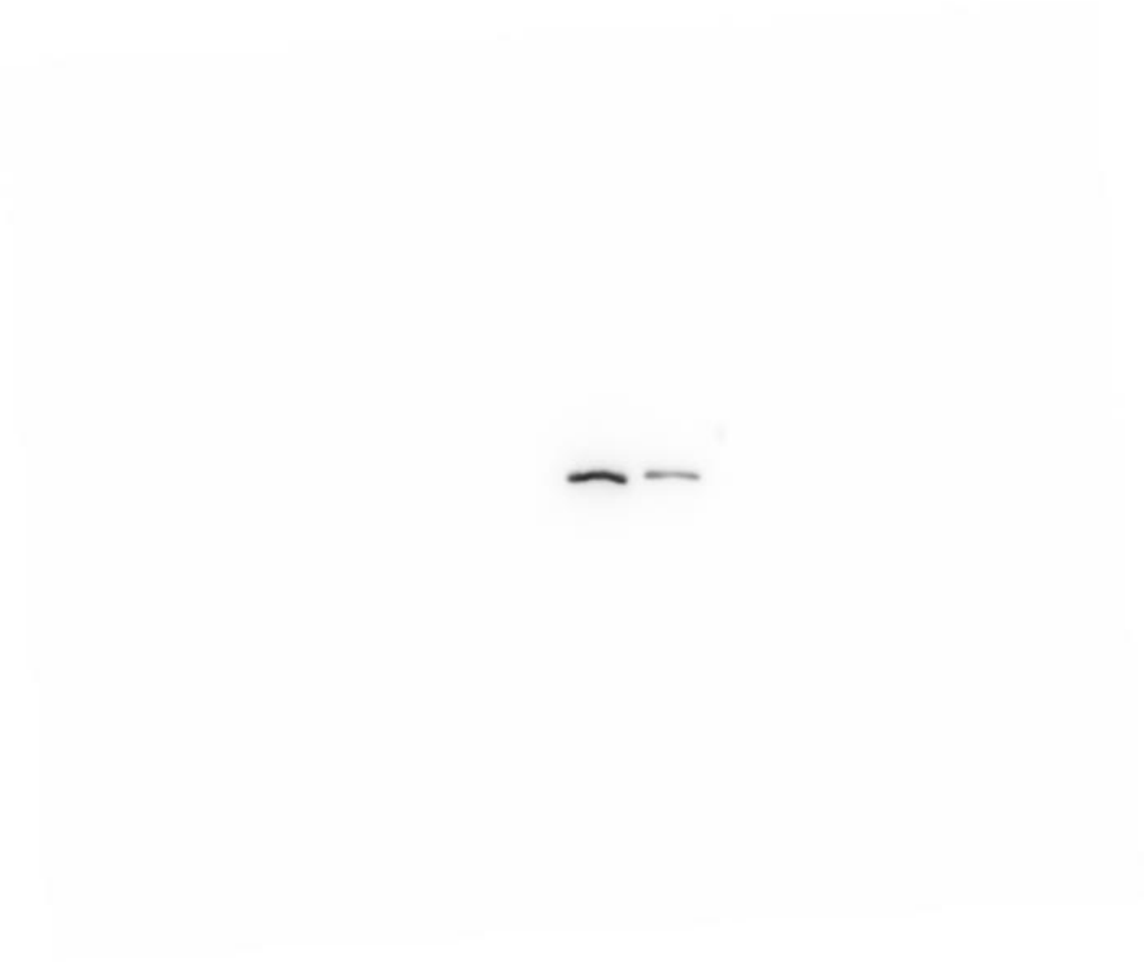

Fig 6H (IB: SCD1 in HLF cells);

Line 1-shCtrl, Line 2-shC12ORF49.

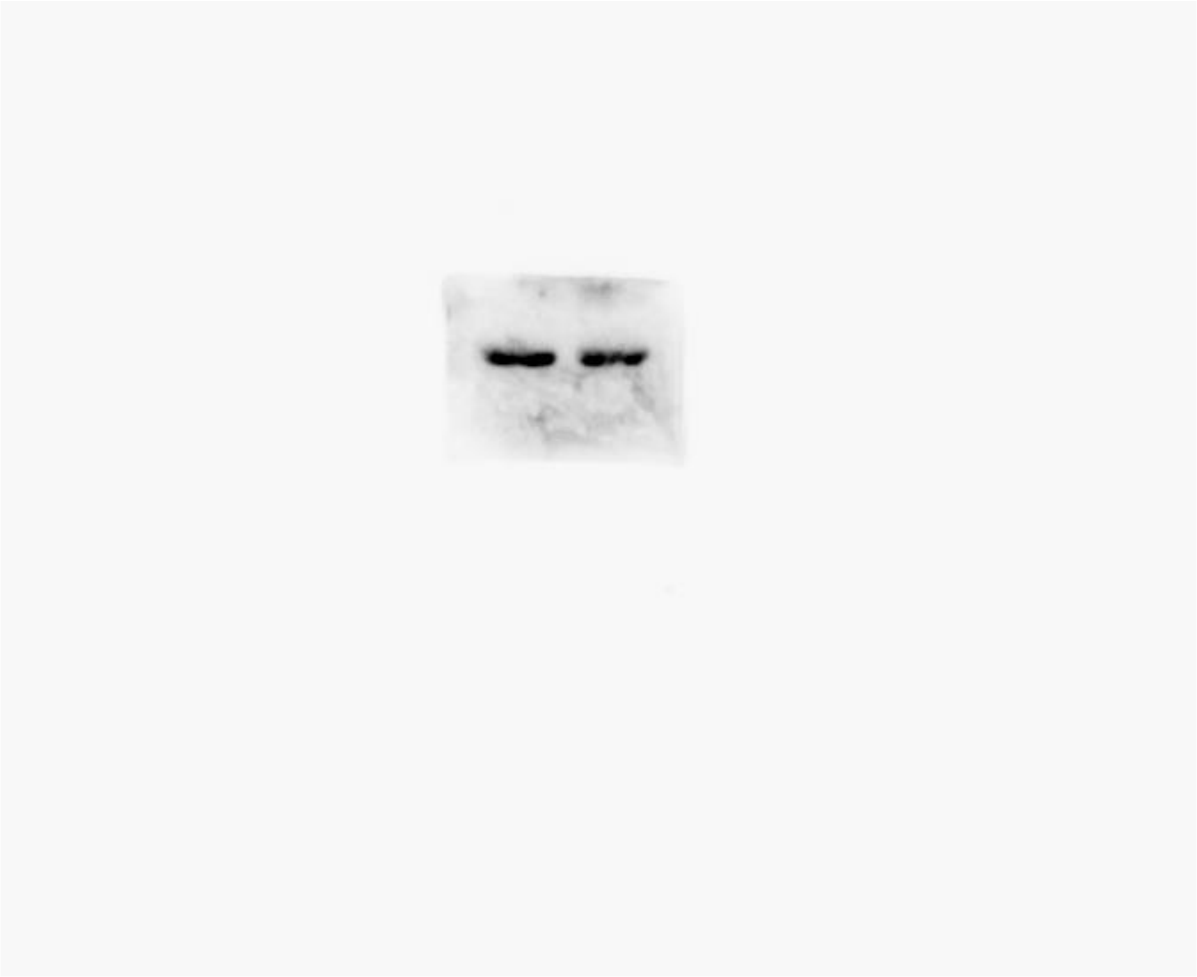

Fig 6H (IB: β-actin in HLF cells);

Line 1-shCtrl, Line 2-shC12ORF49.

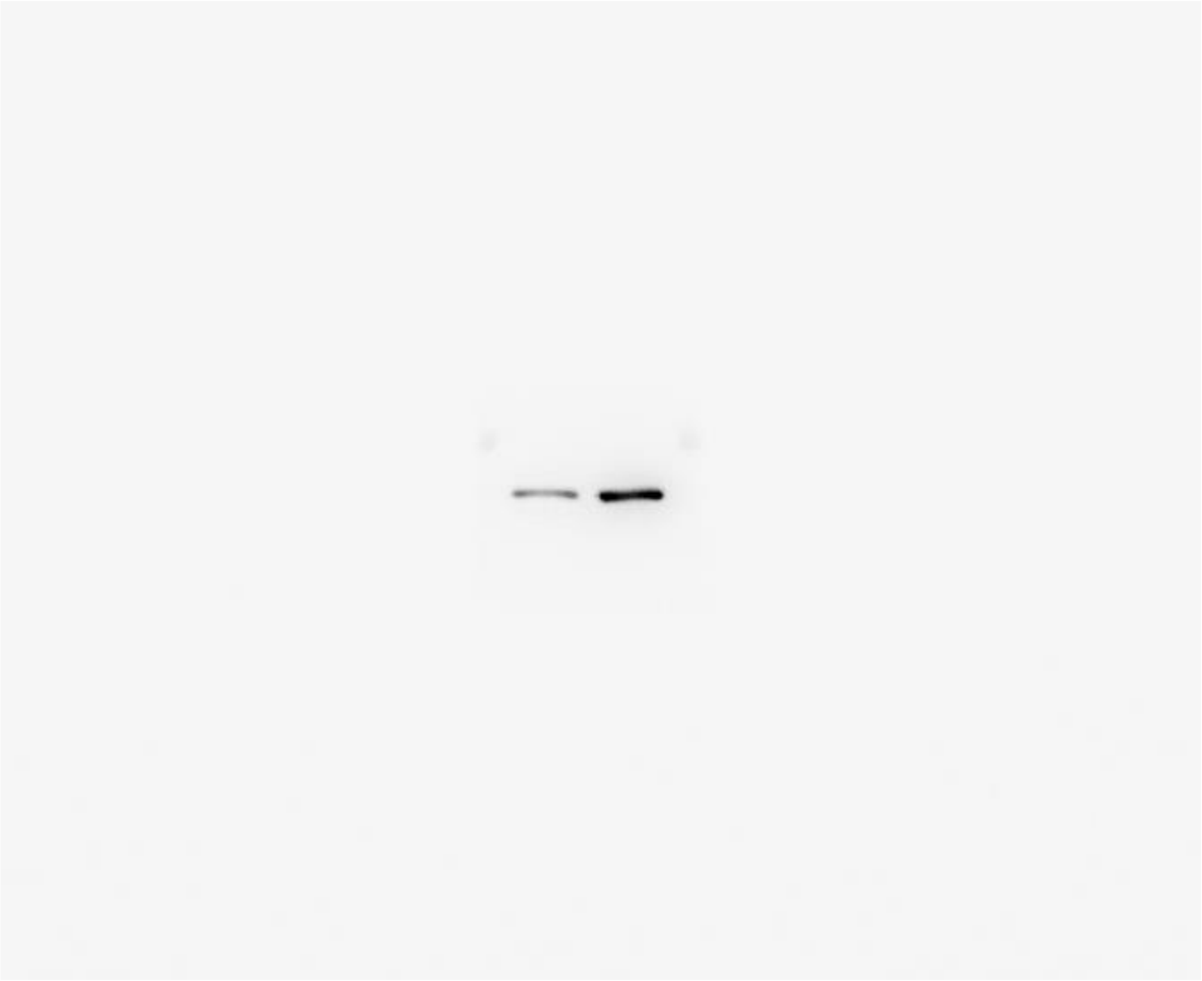

Fig 6H (IB: SCD1 in SNU-354 cells);

Line 1-EV, Line 2-C12ORF49.

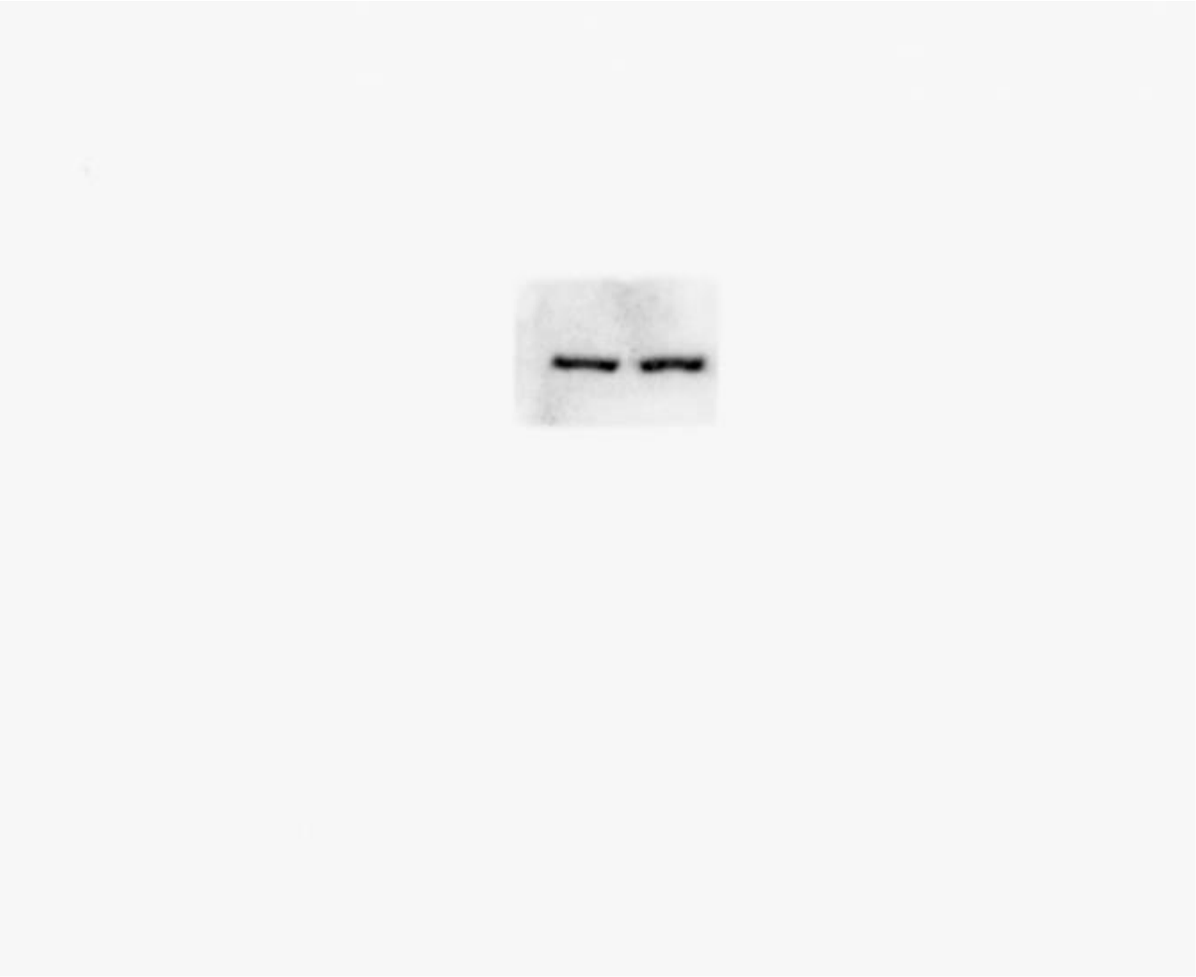

Fig 6H (IB: β-actin in SNU-354 cells);

Line 1-EV, Line 2-C12ORF49.

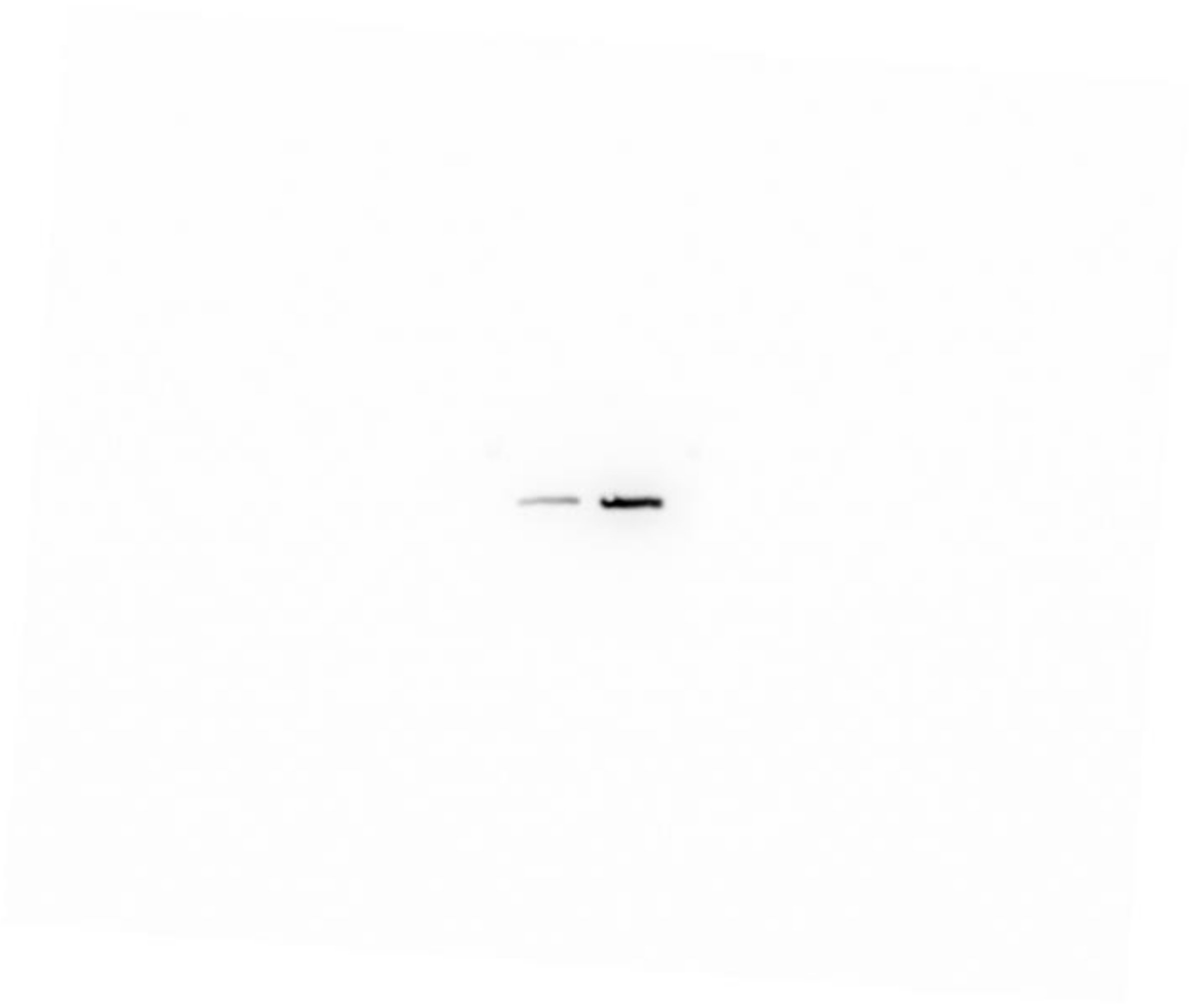

Fig 8G (IB: C12ORF49 in HLF cells)

Line 1-sensitive, Line 2-resistance.

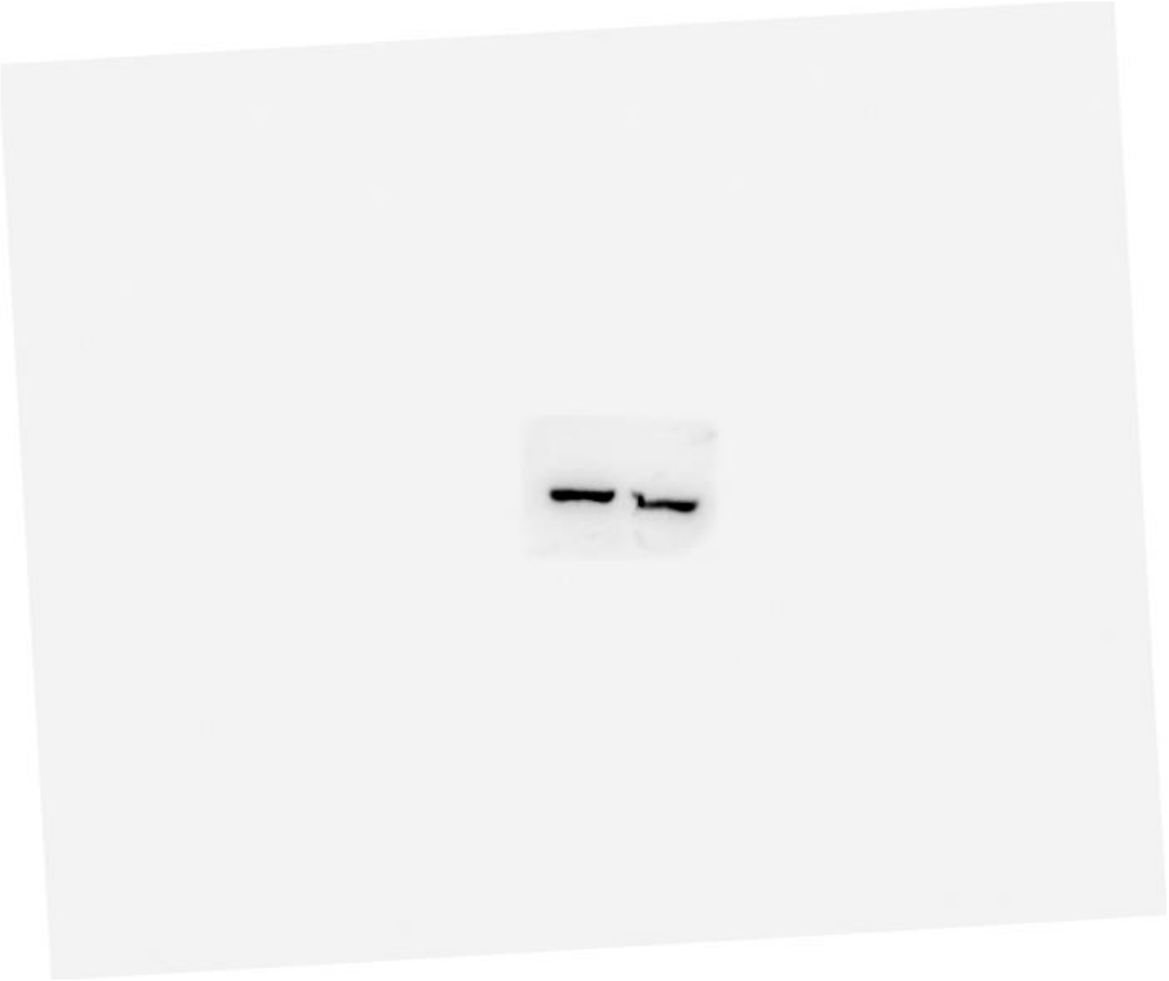

Fig 8G (IB: β-actin in HLF cells);

Line 1-sensitive, Line 2-resistance.

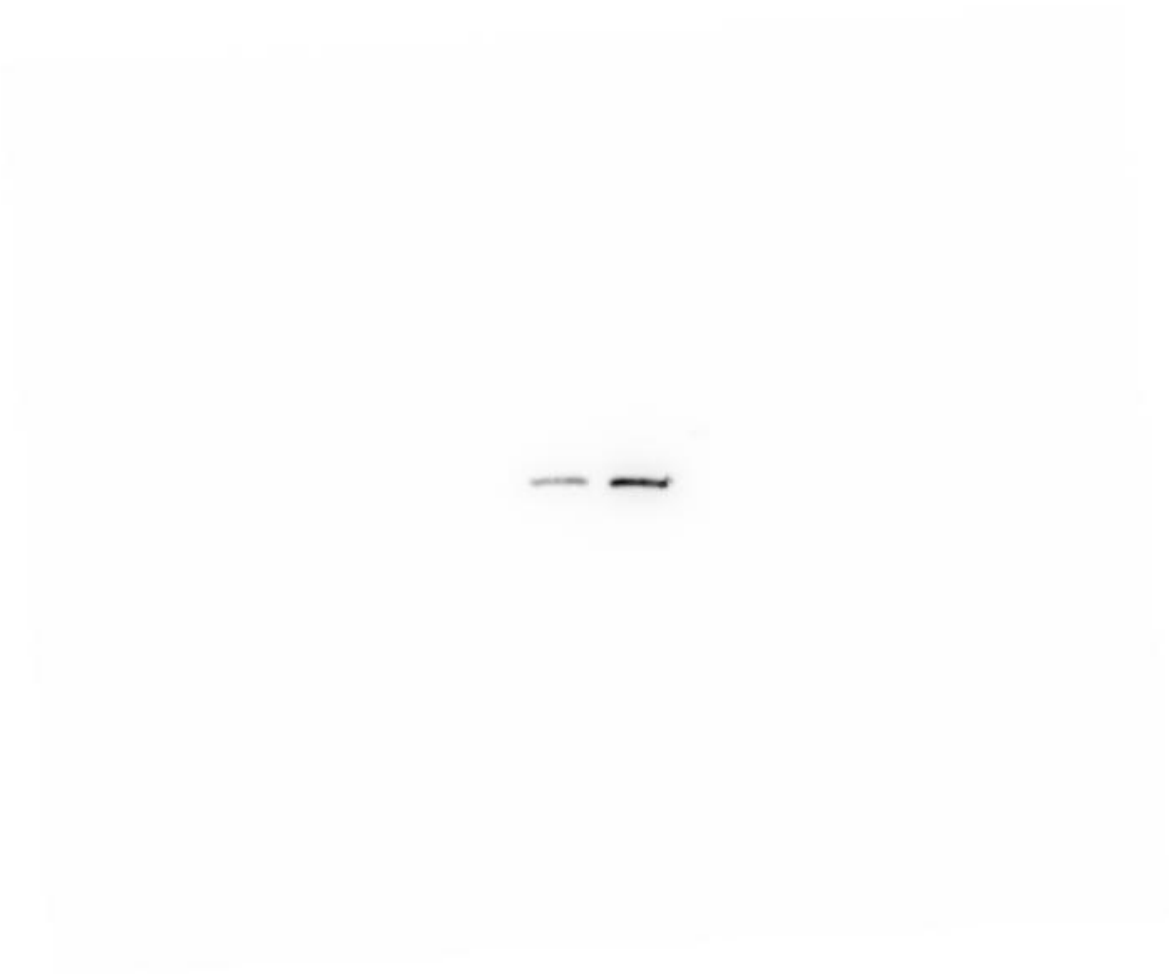

Fig 8G (IB: C12ORF49 in HLE cells);

Line 1-sensitive, Line 2-resistance.

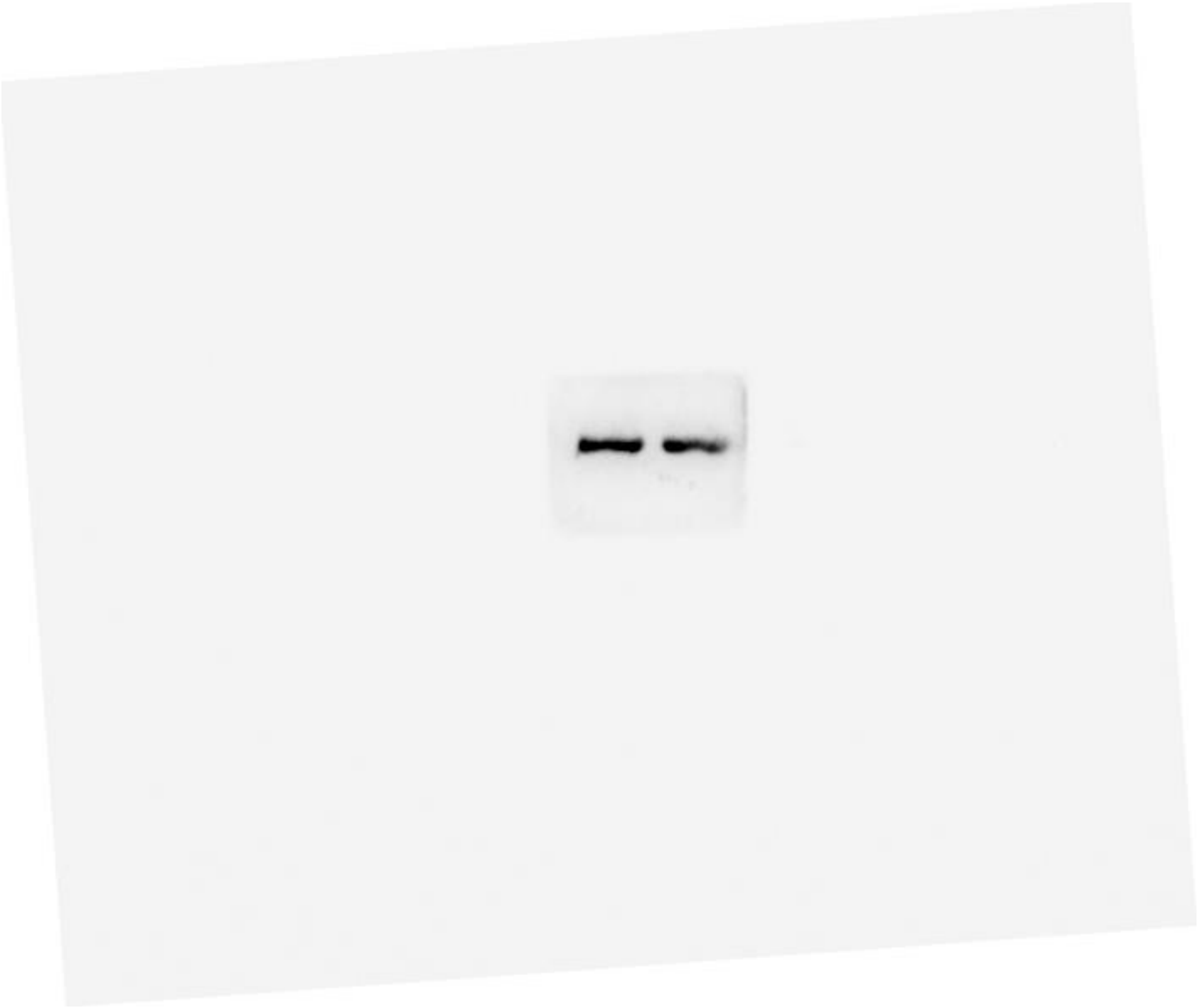

Fig 8G (IB: β-actin in HLE cells);

Line 1-sensitive, Line 2-resistance.
